# C_70_ Fullerene Cage as a Novel Catalyst for Efficient Proton Transfer Reactions between Small Molecules: A Theoretical study

**DOI:** 10.1038/s41598-019-46725-4

**Published:** 2019-07-23

**Authors:** Pradeep R. Varadwaj, Arpita Varadwaj, Helder M. Marques

**Affiliations:** 1 Department of Chemical System Engineering, School of Engineering, The University of Tokyo 7-3-1, Hongo, Bunkyo-ku 113-8656 Japan; 2 The National Institute of Advanced Industrial Science and Technology (AIST), Tsukuba, Ibaraki 305-8560 Japan; 3 Molecular Sciences Institute, School of Chemistry, University of the Witwatersrand, Johannesburg, 2050 South Africa

**Keywords:** Computational chemistry, Electronic structure

## Abstract

When acids are supplied with an excess electron (or placed in an Ar or the more polarizable N_2_ matrix) in the presence of species such as NH_3_, the formation of ion-pairs is a likely outcome. Using density functional theory and first-principles calculations, however, we show that, without supplying an external electron or an electric field, or introducing photo-excitation and -ionization, a single molecule of HCl or HBr in the presence of a single molecule of water inside a C_70_ fullerene cage is susceptible to cleavage of the σ-bond of the Brønsted-Lowry acid into X^−^ and H^+^ ions, with concomitant transfer of the proton along the reaction coordinate. This leads to the formation of an X^−^···^+^HOH_2_ (X = Cl, Br) conjugate acid-base ion-pair, similar to the structure in water of a Zundel ion. This process is unlikely to occur in other fullerene derivatives in the presence of H_2_O without significantly affecting the geometry of the carbon cage, suggesting that the interior of C_70_ is an ideal catalytic platform for proton transfer reactions and the design of related novel materials. By contrast, when a single molecule of HF is reacted with a single molecule of H_2_O inside the C_70_ cage, partial proton transfers from HF to H_2_O is an immediate consequence, as recently observed experimentally. The geometrical, energetic, electron density, orbital, optoelectronic and vibrational characteristics supporting these observations are presented. In contrast with the views that have been advanced in several recent studies, we show that the encaged species experiences significant non-covalent interaction with the interior of the cage. We also show that the inability of current experiments to detect many infrared active vibrational bands of the endo species in these systems is likely to be a consequence of the substantial electrostatic screening effect of the cage.

## Introduction

Proton transfer is at the heart of acid-base chemistry, ubiquitous in many chemical systems and relevant to a wide range of disciplines^1–3^. Partial (or facile) proton transfer from acids occurs when the acidic species is solvated by several H_2_O molecules; this is increasingly recognized for the acids HX (X = Cl, Br, I) that experience dissociation near 0 K in (H_2_O)_*n*_ with *n* ≥ 4.

How individual Brønsted-Lowry acids HX (X = F, Cl, Br, I) interact with Brønsted-Lowry bases such as NH_3_ in an aqueous environment has been studied for decades^1–3^. More recently, interest has developed in exploring how they behave in different environments, such as in the inside of the C_60_ and C_70_ fullerene cages, for example^4–13^. While the underlying chemistry of these interactions plays a central role in the development of acid-base chemistry in the gas, liquid and solid states^14–16^, an understanding of them in various environments, of the processes involved, and their energetics^17^, has proven very useful in the design of new materials^17,18^. This has attracted the interest of scientists working in diverse areas of chemistry, physics, materials and medicinal science, and drug discovery.

A single molecule of HCl, when placed in close proximity to a single molecule of NH_3_, does not experience proton transfer to form the NH_4_^+^···Cl^−^ ion-pair. This is probably because proton transfer from HCl to NH_3_, followed by the creation of the NH_4_^+^ and Cl^−^ conjugate acid-base pair, requires more than 120 kcal mol^−1^ in the gas phase^19^. It is also consistent with the observations of the familiar acid-base behavior in solution^20^. Ordinarily, the HX and NH_3_ (or H_2_O) molecules prefer to form hydrogen bonded complexes^1^; the strength of these bonds varies between −3 and −10 kcal mol^−1^ ^21,22^.

The formation of an NH_4_^+^···Cl^−^ ion-pair is possible, but requires the assistance of local environmental effects, such as collision with other molecules or interactions with excess electrons, ions, or even photons, to trigger proton transfer from the acid to the base. This has been demonstrated experimentally using photoelectron spectroscopy^19,23^. Attempts to generate the ion-pair using an applied electric field and to delineate the underlying mechanisms involved have also discussed^22,24^.

Does such a facile proton transfer process occur between the molecular Brønsted-Lowry acid and the molecular Brønsted-Lowry base when HCl is replaced by HF or HBr, and NH_3_ is replaced by a poorer base such as H_2_O, without supplying an external electron or an Ar/N_2_ matrix environment? The answer is certainly “no”. Is there any simple strategy to initiate proton transfer between H_2_O and HX (X = Cl, Br), leading to the transformation of isolated H_2_O···HX dimers into H_2_OH^+^···^−^X ion-pairs, accompanied by a modification of their geometric, electronic, orbital, optical and vibrational properties?

In this contribution, we answer this fundamentally important question using theoretical methods and show that the formation of the H_2_OH^+^···^−^X (X = C, Br) conjugate ion-pairs is indeed possible when a single H_2_O molecule and a single molecule of either HCl or HBr are accommodated inside a C_70_ cage.

Is the C_70_ cage interior catalytic? Or, is it hydrophobic? We show that it is not at all hydrophobic, but largely catalytic. Inside the interior environment of C_70_ the H_2_O becomes significantly more basic compared to its isolated counterpart. This promotes dissociation of the acids HX (X = Cl, Br) into X^−^ and H^+^ ions. Proton transfer along the O···H hydrogen bond leads to the formation of the H_2_OH^+^···^−^X@C_70_ (X = Cl, Br) ion-pairs. We examined the role played by the cage in facilitating this process and also address the question of whether the resulting species formed between H_2_O and HX is unaffected by (or innocent of) the cage environment. This basic question has been addressed in a number of experimental studies^6,7,11,16^ comprising endohedral fullerene systems with diverse guest species, H_2_O···HF^16^ and others (H_2_, (H_2_)_2_, He, He_2_, H_2_O, HF, H_2_O···H_2_O^11^)^7,8^, but many misleading views have been advanced. If the guest(s) is(are) not innocent, does the intermolecular interaction of the guest with the cage interior modify their fundamental geometric, electronic and vibration properties? What are the vibrational bands that should be taken into account for rationalizing whether the guest molecule is inert or electroactive inside the cage? Are these vibrational bands affected by the dipolar screening effect of the cage, thereby preventing their experimental observation? Is the proton transfer feature associated with the dimers in C_70_ comparable to what might be inferred from the geometries of the same isolated dimers in the first excited state, or in their anionic ground states?

A recent experimental observation^16^ reports that the H_2_O···HF dimer entrapped inside a C_70_ cage experiences a shortening of the O···H hydrogen bond distance and as a consequence the HF bond is elongated. Challenging this interpretation, Jaroš and coworkers^25^ argued that the elongation of the HF bond is a consequence of lone-pair···π_cage_ charge transfer delocalization; hence the shortening of the O···H bond distance is a result of the increased acidity of the HF molecule inside C_70_. What then causes the O···H hydrogen bond distance in the endohedral H_2_O···HF dimer to decrease? We answer these questions below.

## Results and Discussion

### Geometries and nature of potential energy surface

**Figure 1:**
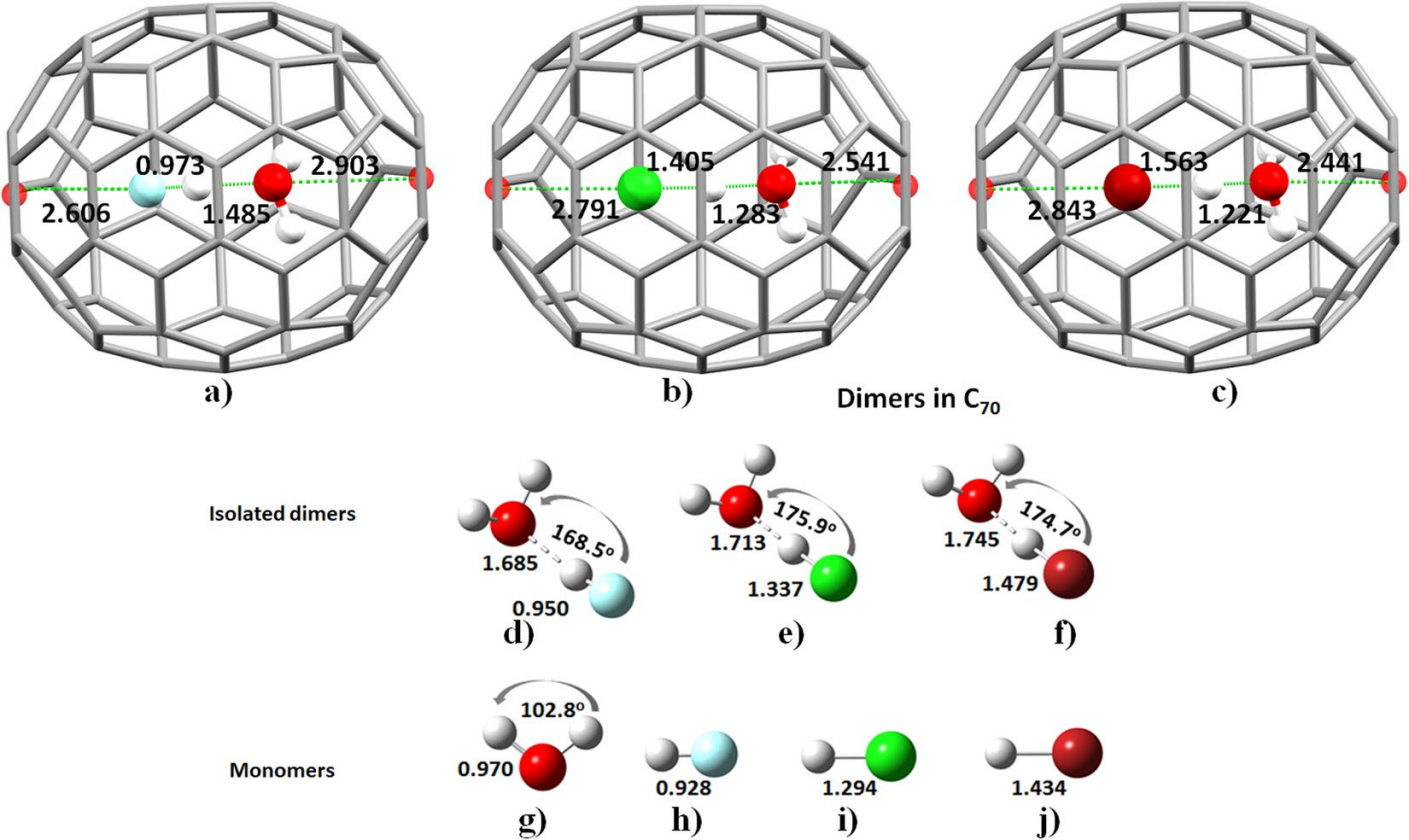
PBE energy minimized geometries of (**a**) H_2_O···HF@C_70_, b) H_2_OH^+^···^−^Cl@C_70_, (**c**) H_2_OH^+^···^−^Br@C_70_, (**d**) H_2_O···HF, (**e**) H_2_O···HCl, (**f**) H_2_O···HBr, (**g**) H_2_O, (**h**) HF, (**i**) HCl, and (**j**) HBr. Selected covalent and intermolecular bond distances are given in Å, and bond angles in deg (see Table S1 for details). The smaller red spheres at the center of five-membered rings of C_70_ represent the centroids.

Figure 1a and d show the energy-minimized geometries of isolated H_2_O···HF and H_2_O···HF@C_70_, respectively, obtained with Gaussian 09^26^; details of the intermolecular bond distances and angles are listed in Table S1.

There are significant differences between the intermolecular geometries of these two systems. On passing from isolated H_2_O**···**HF to H_2_O**···**HF@C_70_, the O**···**H hydrogen bond distance is significantly contracted by 0.200 Å, the H–F covalent bond distance is elongated by 0.023 Å and the O–H bond in H_2_O is elongated by 0.005 Å. The contraction of the O**···**H intermolecular hydrogen bond suggests that it is strengthened, whereas the H–F and O–H bonds are weakened inside C_70_. The latter observations concur with the IUPAC recommendations for identifying hydrogen bonding^27^. The contraction of the O**···**H hydrogen bond leads to the immediate conclusion that the bond gains stability upon encapsulation, possibly indicative of a gain in partial covalent character. This is in agreement with experiment (Fig. S1) and with MP2 calculations, in which, the latter shows that the O**···**F (2.496 Å) distance decreases by 5.5%, whereas the H–F bond distance increases by 2.1%^16^. Other studies reported similar geometrical changes for the H_2_O**···**H_2_O and HF**···**HF dimers inside C_70_^28^.

As found for isolated H_2_O···HF, the isolated H_2_O···HCl and H_2_O···HBr dimers are also hydrogen bonded (Fig. 1e,f). Their encapsulation inside the C_70_ cage, however, caused the bonds in HCl and HBr to stretch sufficiently such that the resulting geometries (Fig. 1b,c) are distinctly different from that of the H_2_O···HF dimer in C_70_. This has required a reorganization energy of −6.76 and −9.34 kcal mol^−1^ for these dimers, respectively, that is significantly larger than the energy of −0.89 kcal mol^−1^ for the H_2_O···HF dimer in C_70_. The relative change in the free energies associated with the geometric reorganization of these three dimers is 2.16, 6.92 and 9.73 kcal mol^−1^, respectively.

The stretching of HCl and HBr bonds in C_70_ is presumably driven by dissociative electron density transfer from H to the Cl and Br atoms, respectively. This has caused cleavage of the bonds in HCl and HBr, and the formation of H^+^ and X^−^ ion pairs in the preliminary step. Although the critical external electric field required for the dissociation of the σ-bonds in isolated HCl and HBr molecules is 510 and 462 MV cm^−1^, respectively, solvation of the proton and the halide anion by water molecules is known to substantially lower the critical electric field by about 300 MV cm^−1^ ^29^. This is the likely scenario inside the cage as the HX bond breaking in the presence of the H_2_O molecule is due to the encapsulation efficiency of the C_70_ cage.

The dissociation of the acids (HCl and HBr) did not enable the H^+^ ion to migrate towards the interior carbon surface of the cage. Rather, the proton moved apart from X^−^ along the reaction (hydrogen-bond) coordinate, a process that involves a simple proton motion between the X^−^ and H_2_O. The bridging proton resides at an intermediate position between X^−^ and O at 1.405 and 1.563 Å from the Cl^−^ and Br^−^ ions, respectively, and was found at 1.283 and 1.221 Å from O(H_2_); the latter distances suggest the formation of the hydronium ion (H_3_O^+^). Clearly, the structure of the bridging proton for these contact ion-pairs, H_2_O–H^+^**···**Cl^−^ and H_2_OH^+^**···**Br^−^, is similar to the structure of the water Zundel ion, (H_2_)O–H–O(H_2_)^+^ ^30^. This is facilitated by the near linearity of the double charge-assisted hydrogen bonds (∠O–H^+^···X^−^ = 172.9° for H_2_O–H^+^**···**Cl^−^ and 164.8° for H_2_O–H^+^**···**Br^−^ in C_70_) and the short distance between the H^+^ and X^−^ ion pairs (*r*(H^+^···X^−^) 1.405 Å for H_2_O–H^+^**···**Cl^−^ and 1.563 Å for H_2_O–H^+^**···**Br^−^). For comparison, a detail of the nature of the geometry of isolated H_2_O···HX (X = F, Cl, Br) dimers in their first excited and in their anionic ground states is given in TEXT S1 of the Supplementary Information.

**Figure 2:**
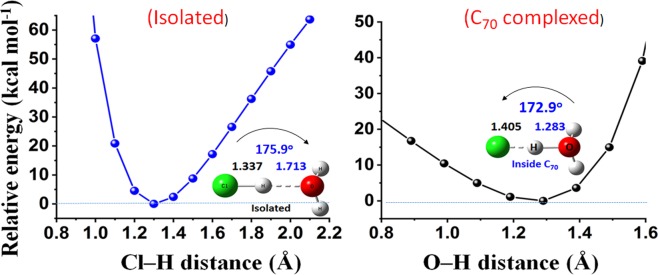
MP2/aug-cc-pVTZ level asymmetric single-well potential energy surface (rigid) of H_2_O**···**HCl (left) and H_2_OH^+^**···**Cl^−^ (right), showing a proton bound to and transferred from the Cl^−^ species, respectively (PBE/6-311 G(d, p) geometry was used for the rigid scan). Bond distances are in Å and the ∠O**···**H–Cl and ∠O**–**H**···**Cl bond angles in deg. An identical result was found for the corresponding H_2_O**···**HBr and H_3_O^+^**···**Br^−^ systems.

The proton transfer from the acids HX was made possible because the cage substantially minimized the potential barrier between HX and H_2_O (Fig. 2). The proton transfer is essentially a one-dimensional process in these systems. The chemical process involved for H_2_O–H^+^**···**X^−^ ion-pair formation is analogous to that previously described by Gutberlet *et al*.^1^. Specifically, it was shown in that study that the successive aggregation of HCl with H_2_O leads to the formation of HCl(H_2_O)_*n*_ hydronium complexes for *n* = 4, involving the dissociated H_3_O^+^(H_2_O)_3_Cl^−^ ion-pair. Ma *et al*. have reported a similar result^31^.

The formation of the hydronium anion inside the C_70_ cage can be understood from the difference between the proton coordinate distances, Δ. This parameter is positive when the H atom is bound to the halogen atom, and negative when the H atom is detached and moves away^32^. For instance, the HCl and HBr bond distances in the monomers are 1.294 and 1.434 Å, respectively (Fig. 1i,j), whereas those in the isolated dimers H_2_O**···**HCl and H_2_O**···**HBr are 1.337 and 1.479 Å, respectively (Fig. 1e,f). These show that isolated H_2_O**···**HF, H_2_O**···**HCl and H_2_O**···**HBr dimers are associated with Δ > 0 (Tables S1 and S2), meaning the halides are covalently bound to the H atoms in these dimers. The same is also true for the H_2_O**···**HF dimer in H_2_O**···**HF@C_70_ (Table S1).

The H_3_O^+^**···**Cl^−^ and H_3_O^+^**···**Br^−^ ion-pairs, on the other hand, are characterized by negative Δ values (Table S2). This signifies that the halide anions are indeed separated from the H^+^ and the latter is bound covalently to H_2_O molecule in these dimers inside C_70_.

### Stabilization energies

We estimated the binding energies of the H_2_O···HF dimer in the absence and in the presence of the C_70_ cage at various levels of theory, including [CCSD(T)/aug-cc-pVTZ], as was done in previous studies on other systems^13,25,28,31^. From the sign and magnitudes of the uncorrected (Δ*E*) and basis set corrected (Δ*E*(BSSE)) binding energies (Table S1), it is quite apparent that the isolated H_2_O**···**HF dimer is less stable (<1 kcal mol^−1^ at all levels of theory) than in H_2_O**···**HF@C_70_; this is accompanied by the O**···**H bond contraction. Our zeroth level symmetry adapted perturbation theory^33^ calculation suggests that the interaction energy of the isolated H_2_O**···**HF dimer can be described in terms of the dissected energy components arising from electrostatics (−15.56 kcal mol^−1^), exchange repulsion (+14.52 kcal mol^−1^), polarization (−6.37 kcal mol^−1^) and dispersion (−2.61 kcal mol^−1^). Its encapsulation inside the C_70_ markedly increases these component energies by 1.44, 1.96, 1.96 and 1.56 times, respectively, thus suggesting that the effect of exchange repulsion is increasingly dominant and is compensated by polarization and dispersive effects.

We have also estimated the Δ*E* of the H_2_OH^+^**···**^−^Cl and H_2_OH^+^**···**^−^Br ion-pairs using a variety of computational approaches; the results are summarized in Table S2. In doing so, the fragments such as OH_3_^+^ and X^−^ (X = Cl, Br) were taken as the two fragments of the complex. As expected, very large values for Δ*E* and Δ*E*(BSSE) resulted for both the complexes, confirming the formation of ion-pairs. Such large values are expected given that complex formation is predominantly electrostatically driven, with a significant transfer of charge between the interacting partners. Binding energies of ion-pairs of similar magnitudes have been reported in many recent studies^34–41^.

### Nature of charge rearrangement and charge transfer

The isolated H_2_O**···**HF, H_2_O**···**HCl and H_2_O**···**HBr dimers have stabilization energies of −8.57, −5.18 and −3.57 kcal mol^−1^ with CCSD(T), respectively, indicative of the presence of weak-to-medium strength hydrogen bonded interactions^34,42^. The charge transfer for these dimers is 0.056, 0.062 and 0.064 *e*, respectively. The result of a second-order perturbation theory analysis of the Fock matrix in natural bond orbital basis^43^ suggests this charge transfer occurs from the oxygen lone-pair orbital to a σ*(H–F) anti-bonding orbital of HX2.

Significant charge rearrangement accompanying encapsulation of the guest dimers is notable in Figs S2–S5. Depending on the charge polarity of a particular atom in the guest, a specific portion of the cage is polarized with an opposite sign so as to facilitate a Coulombic attraction between them. Because of this, the charge-transfer increases significantly with cage residence, suggesting a further transfer of charge between the monomers inside the host. This is evidenced by the increase in the partial charge of the H atom by 0.0044, 0.1080 and 0.2024 *e* for H_2_O**···**HF@C_70_, H_2_OH^+^**···**^−^Cl@C_70_ and H_2_OH^+^**···**^−^Br@C_70,_ respectively, compared to those in the isolated dimers (Fig. S2). The net charge transfers between the C_70_ host and the H_2_O**···**HF, H_2_O**···**HCl and H_2_O**···**HBr guests is 0.107, 0.094 and 0.102 *e* in the encapsulated complexes; the absence of a trend in the charge transfer values is due to the integration error. This result suggests that all the three individual dimers in C_70_ are Mulliken inner type complexes^34,42^.

Arrhenius theory suggests that when the acid HA reacts with the base B(H_2_O)_*n*_ this causes the formation of neutral H_2_O molecules and a charge-neutral salt, *n*(AH) + B(H_2_O)_*n*_ ⇌ B^*n*+^(A^−^)_*n*_ + *n*(H_2_O)^44^. This widely applicable concept is true for the isolated H_2_O**···**HF, H_2_O**···**HCl and H_2_O**···**HBr dimers that are found to be electrically neutral. However, this is not so when these dimers are enclosed inside C_70_. Our result suggests that the H_2_O**···**HF, H_2_O**···**HCl and H_2_O**···**HBr dimers are no longer electrically neutral inside C_70_ (Figs S3–S5), in agreement with previous reports on non-fullerene-based systems (for example^45^). Although the former is a hydrogen bonded dimer and the latter two are locally ion-pairs inside C_70_, the net fractional charge conferred on the guests [H_2_O**···**HF], [H_2_OH^+^**···**^−^Cl] and [H_2_OH^+^**···**^−^Br] is −0.077, −0.030 and −0.023 *e*, respectively. The net fractional charge on the C_70_ is +0.077, +0.030 and +0.023 *e* for H_2_O**···**HF@C_70_, HOH^+^**···**^−^Cl@C_70_ and HOH^+^**···**^−^Br@C_70_, respectively. These results signify that each of the dimers inside C70 transforms in its entirety as a conjugate anionic base and the C_70_ host transforms as a conjugate cationic acid (C_70_^+^). Clearly, the charge redistribution (Figs S3–S5) accompanying the formation of the endohedral systems enforces the host and the guest to serve as a separate conjugate acid-base pair for all the three systems. Because of this feature, one might conclude that the H_2_O**···**HF@C_70_ dimer is a single ion-pair (e.g. ([H_2_O**···**HF]^−0.077*e*^[C_70_^+0.077*e*^])), whereas the H_2_OH^+^**···**^−^Cl@C_70_, H_2_OH^+^**···**^−^Br@C_70_ dimers are double ion-pairs. This might explain why the C_70_ cage interior is ideal for the dissociation of a Brønsted acid in the presence of H_2_O, accompanied by proton transfer from the acid to H_2_O and solvation of the charged products. Previous studies have shown that *anionic* C_60_-mediated reduction reactions involving H_2_O are very promising for proton transfer processes^46^; similar results have been reported for non-fullerene based compounds as well^47,48^.

### Effect of electrostatic screening on the polarity of the dimers

The formation of the ion pairs inside the C_70_ cage is evidence of the unusually strong charge-assisted hydrogen bond energies (Table S2). It was therefore expected that the polarity of the ion-pair would increase significantly. This is in agreement with a previous study^23^; the dipole moment of the H_3_N**···**HCl system increased from 4.15 to 9.82 D upon the intermolecular proton transfer that enabled the formation of the Cl^−^**···**HNH_2_^+^ ion-pair salt. However, we did not notice any such enhancement in the dimer dipole moments for either H_2_O**···**HF@C_70_, H_2_OH^+^**···**^−^Cl@C_70_, or H_2_OH^+^**···**^−^Br@C_70_, even though this is a common characteristic on the formation of binary complexes of Mulliken inner and outer types^34^. The anomaly could thus be attributed to the effect of the cage interior that effectively screens the dipolar electric field of an entrapped species (irrespective of whether this is an ion-pair, a simple hydrogen bonded dimer, or simply an H_2_O molecule).

To substantiate this, our calculated values of the dipole moments of H_2_O**···**HF@C_70_, H_2_OH^+^**···**^−^Cl@C_70_, H_2_OH^+^**···**^−^Br@C_70_ were 0.70, 1.03 and 1.07 D, respectively. These are significantly smaller (15.6, 18.0 and 18.5%) than the dipole moments of the H_2_O**···**HF, H_2_OH^+^**···**^−^Cl and H_2_OH^+^**···**^−^Br dimers computed at their geometries after removing the C_70_ cage, *viz*., 4.50, 5.73 and 5.77 D, respectively. These latter values, as expected, are larger than those of isolated dimers H_2_O**···**HF, H_2_O**···**HCl, and H_2_O**···**HBr, respectively, *viz*. 3.98, 4.42, and 4.13 D, demonstrating the presence of dipolar screening effect developed by the intrinsic electric field of the C_70_.

### Effect of encapsulation on the emergence of significant intermolecular interactions: A QTAIM rationalization

**Figure 3:**
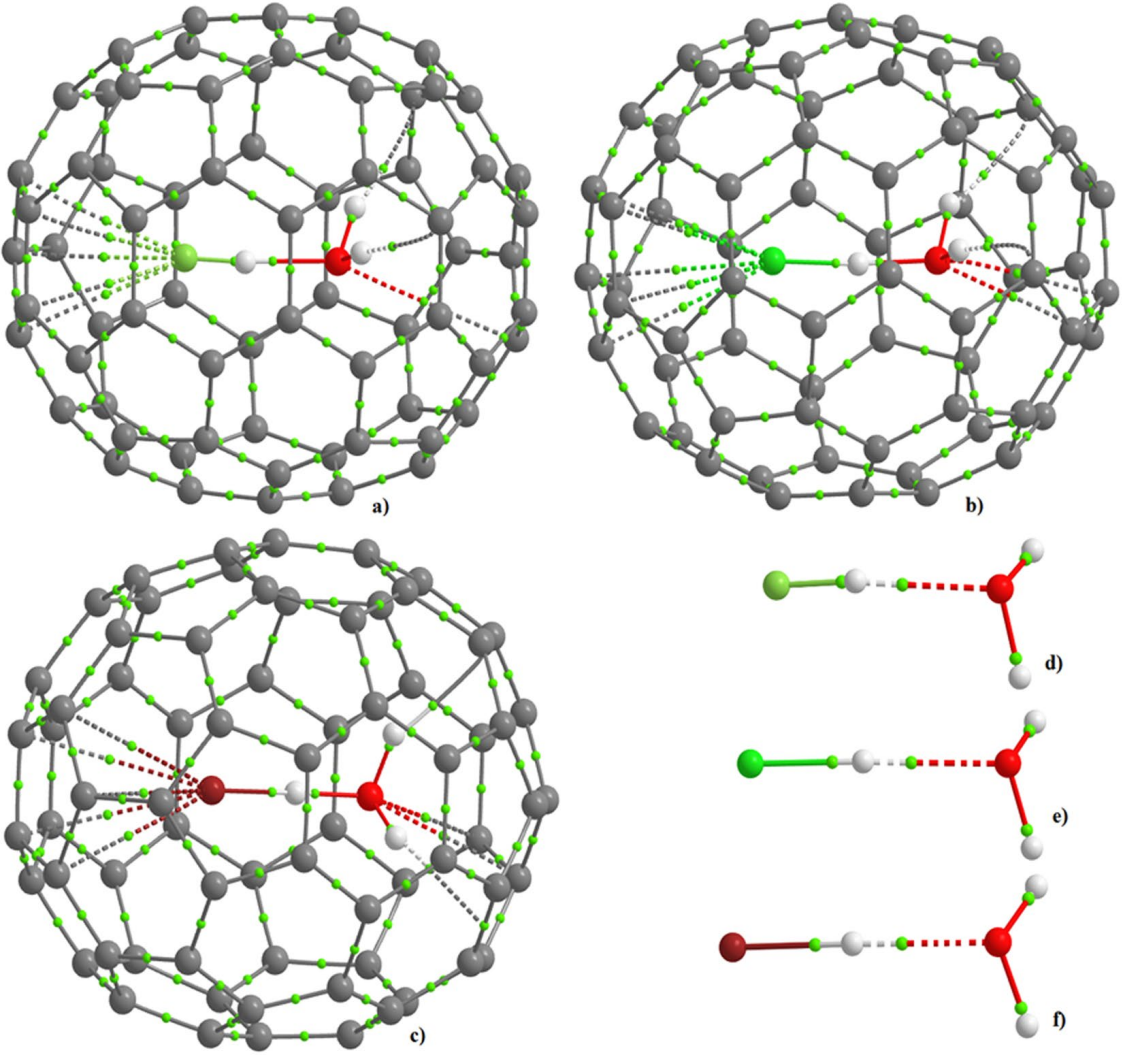
PBE level molecular graph for (**a**) H_2_O**···**HF@C_70_, (**b**) H_2_OH^+^**···**^−^Cl@C_70_, and (**c**) H_2_OH^+^**···**^−^Br@C_70_, (**d**) H_2_O**···**HF, (**e**) H_2_O**···**HCl and (**f**) H_2_O**···**HBr. Each hydrogen atom in H_2_O is involved in a single hydrogen bonding interaction with carbon, whereas each halogen atom is involved in a cone of interaction with the neighboring 5-membered aromatic ring of the C_70_ cage interior; these are revealed by the bond path and bond critical point topologies of the charge density. Bond paths are shown in dotted and solid lines in atom color, and bond critical points as tiny spheres in green. The intermolecular interaction between the host and the guest for each system is elucidated by the bond path topologies shown as solid and dotted lines in atom color.

A fundamental question in endohedral systems is whether the guest species is innocent inside the cage interior, or whether it experiences significant intermolecular interactions^49^. In several studies reported over the last seven years^6,11,16^, it was shown that a guest species, such as H_2_O, residing near the center of the cage, is not involved either in hydrogen bonding or in π**···**H interactions with the interior carbons of the five- and six-membered rings of the C_60_ and C_70_ cages. We examined the bond critical point (bcp) and bond path topologies of potential bonding interactions (Fig. 3) using Bader’s quantum theory of atoms in molecules (QTAIM)^50,51^. We found that the charge density, *ρ*_b_, at the O**···**H bond critical point increased from 0.047 to 0.078 a.u. on passing from H_2_O**···**HF to H_2_O**···**HF@C_70_, with a concomitant change in the Laplacian of the charge density (∇^2^*ρ*_b_) of +0.143 to +0.179 a.u. These signatures are consistent with the contraction of the O**···**H bond in H_2_O**···**HF upon its encapsulation, suggesting that there is no H_3_O^+^**···**F^−^ ion-pair formed inside the C_70_ cage, as claimed previously^16^.

In H_2_O**···**HF@C_70_, the H atoms of the H_2_O molecule are found not to be inert. Each H atom in H_2_O is engaged in a C_π_**···**H–O hydrogen bonding interaction with an adjacent carbon of the C_70_ cage (Fig. 3). The *ρ*_b_, ∇^2^*ρ*_b_, and the total energy density (*H*_b_) at the C_π_**···**H–O bcps are calculated to be 0.012, +0.044 and +0.0021 a.u., respectively. In addition, the lone-pair bonding orbital of the O atom is observed to act as a donor of electron density, which is evidenced by the formation of O**···**C interactions for which *ρ*_b_, ∇^2^*ρ*_b_ and *H*_b_ are approximately 0.0083, +0.031, and +0.0010 a.u., respectively.

There is a similar involvement of the H atoms of H_2_O with the carbon cage in H_2_OH^+^**···**^−^Cl@C_70_ and H_2_OH^+^**···**^−^Br@C_70_. For these two cases, the C_π_**···**H–O interactions are somewhat stronger and are very asymmetric. This can be seen from the values of *ρ*_b_, ∇^2^*ρ*_b_ and *H*_b_, in which, the two C_π_**···**H–O hydrogen bonds in H_2_OH^+^**···**^−^Cl@C_70_ have *ρ*_b_ = 0.022 and 0.019 a.u., ∇^2^*ρ*_b_ = 0.063 and 0.0717 a.u., and *H*_b_ = +0.0017 and +0.0024, respectively. The corresponding values for H_2_OH^+^**···**^−^Br@C_70_ are 0.020 and 0.025, +0.067 and +0.079, +0.0023 and +0.0010 a.u., respectively. Likewise, the O**···**C interactions with the cage in H_2_OH^+^**···**^−^Cl@C_70_ and H_2_OH^+^**···**^−^Br@C_70_ are also found to be strengthened.

In each of these systems, the halogen is involved in a five-center, six bond topology, forming a cone of intermolecular interactions reminiscent of that found for cyclopentadienyl and substituted dienyl bonding to titanium^52^. Such a topology is of significance in the understanding of non-classical metal-to-saturated-carbon atom interactions. Even though the fluorine in H–F resides at a shorter distance from the interior carbon wall of the C_70_ cage compared to the chlorine and bromine atoms in H–X (X = Cl, Br), it is comparatively more weakly bound to the carbons of the five-membered aromatic ring which it faces. The attractive engagement between the F in the H–F fragment and the C_5_ ring of the cage can be recognized as π**···**σ_hole_ (negative) interactions^53^, whereas for the other two this could be standard π**···**X^−^ (X = Cl, Br) interactions. The strength of the C_π_**···**X interactions is gauged from the values of *ρ*_b_: 0.010 a.u. for C**···**F; 0.014 for C**···**Cl; and 0.015 a.u. for C**···**Br. The ∇^2^*ρ*_b_ values for the corresponding interactions are +0.048, +0.0053 and +0.055 a.u., respectively, indicative of closed-shell interactions.

### Nature of intermolecular interactions: A reduced density gradient and independent gradient model perspective

**Figure 4:**
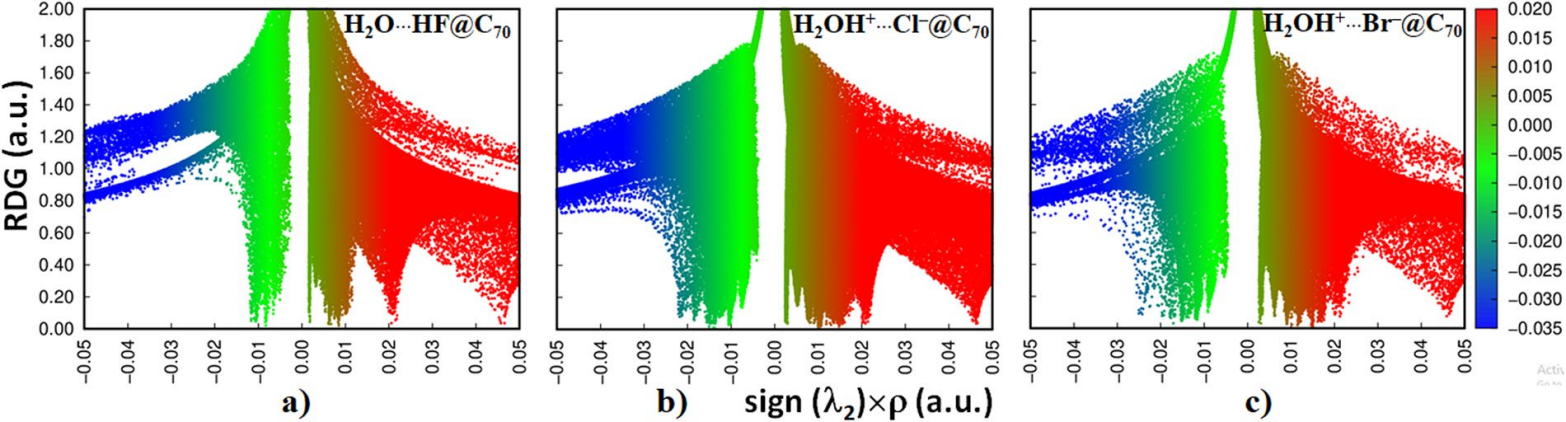
PBE level sign(*λ*_2_) × *ρ vs*. RDG 2D plots (0.5 a.u.) for (**a**) H_2_O**···**HF@C_70_, (**b**) HOH^+^**···**^−^Cl@C_70_, and c) HOH^+^**···**^−^Br@C_70_, showing significant attractive interactions between the host and the guest species.

Reduced Density Gradient Non-Covalent Index (RDG-NCI)^54^ and Independent Gradient Model (IGM)^55,56^ analyses suggest that regardless of the nature of the guest species, they are involved in an attractive engagement with the interior carbons of the C_70_ cage. This is true not only for the isolated guest molecules (viz. H_2_O and HX (X = F, Cl, Br)), but also for the dimers formed by them. Figure 4 manifests this for the three endohedral systems, revealed using the sign(*λ*_2_) × *ρ vs*. RDG plots of the RGD-NCI analysis (*λ*_2_ is the second eigenvalue of the Hessian second derivative charge density matrix). There are several spikes in each of these plots. The green (for H_2_O**···**HF@C_70_) and bluish-green spikes (for HOH^+^**···**^−^Cl@C_70_ and HOH^+^**···**^−^Br@C_70_) are prominent in the sign(*λ*_2_) < 0 regions, and the red ones in the sign(*λ*_2_) > 0 regions. The first represents attraction between the host and guest species and the second corresponds to repulsion in the RDG picture. Although the nature of the (green- and bluish-green) spikes are consistent with the level of attraction observed between the host and the guest species, it is largest for HOH^+^**···**^−^Br@C_70_ compared to the other two (i.e., in the order H_2_O**···**HF@C_70_ < HOH^+^**···**^−^Cl@C_70_ < HOH^+^**···**^−^Br@C_70_).

**Figure 5:**
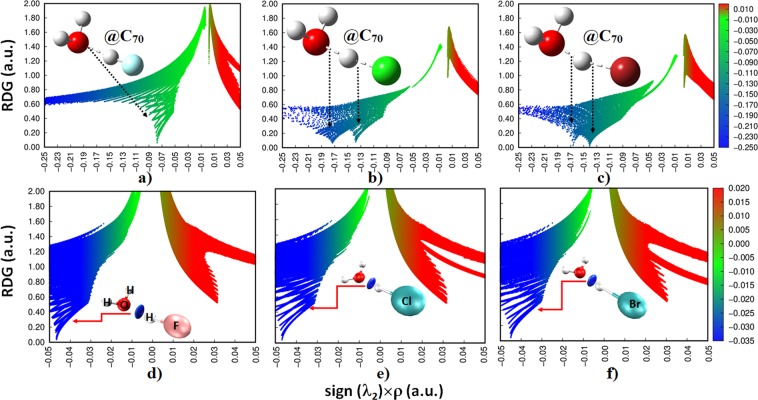
PBE level sign(*λ*_2_) × *ρ vs*. RDG plots (0.5 a.u.) for the encaged dimers: (**a**) H_2_O**···**HF@C_70_, (**b**) HOH^+^**···**^−^Cl@C_70_, and (**c**) HOH^+^**···**^−^Br@C_70_. Shown are also the corresponding plots for isolated dimers: (**d**) H_2_O**···**HF, (**e**) H_2_O**···**HCl and (**f**) H_2_O**···**HBr.

Figure 5 compares the sign(*λ*_2_) × *ρ vs*. RDG 2D plots of H_2_O**···**HF@C_70_, HOH^+^**···**^−^Cl@C_70_ and HOH^+^**···**^−^Br@C_70_ (a-c, top) with those of the isolated H_2_O**···**HF, H_2_O**···**HCl and H_2_O**···**HBr dimers (d-f, bottom). Two distinct features are reminiscence of these plots. In d-f), the O**···**H noncovalent interactions between the H_2_O and HX monomers are characterized by a single blue spike in the range −0.05 a.u. < sign(*λ*_2_) × *ρ* < −0.045 a.u. for all isolated dimers, which are fingerprints of attractive interactions. The nature of the charge density and the location of the spikes reflected in these plots are marginally different from one another, consistent with the trend in the stability of the hydrogen bonds found in the dimers (H_2_O**···**HF > H_2_O**···**HCl > H_2_O**···**HBr).

The encapsulation of H_2_O**···**HF led the equilibrium O**···**H distance to contract, which is consistent with the enhanced electron density in the critical bonding region. Hence the peak position of the RDG spike shifted significantly to a high-density region, −0.07 a.u. < sign(*λ*_2_) × *ρ* < −0.08 a.u. (Fig. 5a). The transfer of the proton from the HX moiety to the H_2_O molecule and subsequent formation of the hydronium ion in the equilibrium geometries of the H_2_OH^+^**···**^−^Cl@C_70_ and H_2_OH^+^**···**^−^Br@C_70_ systems explains why there are two RGD spikes observed in Fig. 5b and c, respectively. These provide evidence that the O**–**H^+^ bond is relatively stronger than the H^+^**···**^−^X (X = Cl, Br) interactions in these two complexes. Nevertheless, the H^+^**···**^−^X interactions are not only predominantly electrostatic, but also contain a non-negligible amount of covalency^57^.

**Figure 6:**
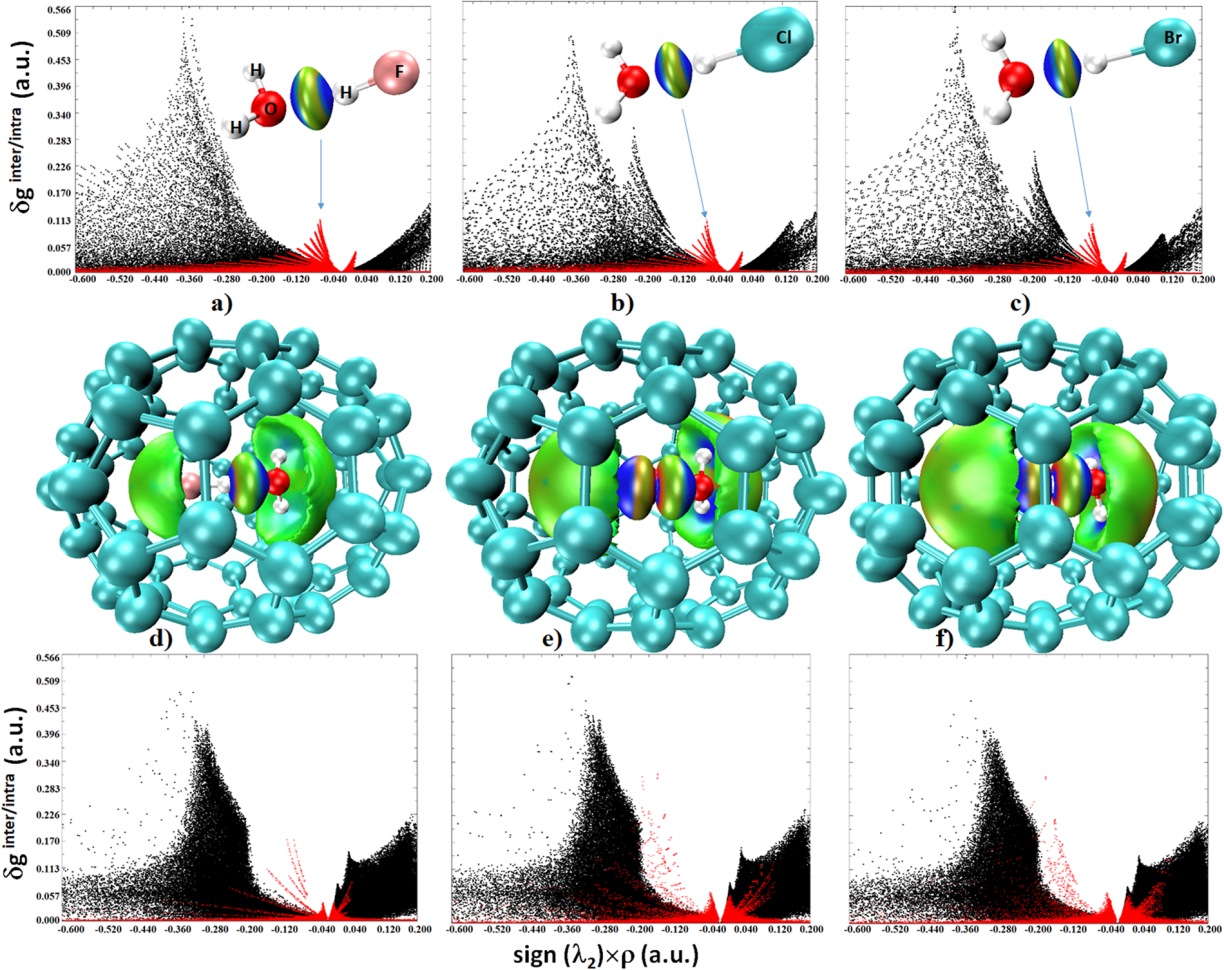
(Top) PBE level IGM isosurface and spike plots for the isolated dimers: (**a**) H_2_O**···**HF, (**b**) H_2_O**···**HCl and H_2_O**···**HBr (0.02 a.u.). (Middle and bottom) PBE level IGM isosurface and spike plots for the encaged species: (**d**) H_2_O**···**HF@C_70_, (**e**) HOH^+^**···**^−^Cl@C_70_, and f) HOH^+^**···**^−^Br@C_70_ (0.01 a.u.).

Figure 6 compares the IGM results for the isolated and encaged systems. This innovative approach automates the identification of intermolecular interactions using actual and pro-molecular electron densities. A descriptor of the model, called δ*g*^inter/intra^ (inter- and intra-molecular contributions), reveals interactions beyond the presence of the bond critical points. As can be seen from a)-c) and d)-f), the isosurfaces (blue, green and mixed color) are centered around the bond critical point regions in the 3D maps of all the isolated and endohedral systems, signifying stabilizing interactions between interacting basins. The broad black spikes of the sign(*λ*_2_) × *ρ vs*. δ*g*^inter/intra^ plots are associated with covalent bonds, appearing in the sign(*λ*_2_) × *ρ* < 0 region and corresponding to high δ*g*^intra^ values. The hydrogen bonds and van der Waals interactions correspond to the spikes at low-density that are associated with low δ*g*^inter^ values. These are represented by two small and sharp spikes (red); one is due to attraction and corresponds to *λ*_2_ < 0 and the other, due to repulsion, corresponds to *λ*_2_ > 0 in reduced gradient density picture. The sign(*λ*_2_) × *ρ vs*. RDG plots for H_2_O**···**HF@C_70_, H_2_OH^+^**···**^−^Cl@C_70_ and H_2_OH^+^**···**^−^Br@C_70_ illustrated in d)-f) of Fig. 6 corroborates the inferences from the RDG analysis (Figs 5 and 6). However, the isosurface plots suggest that attraction between the guest and the fullerene C_70_ cage is significantly dispersed, and is in excellent agreement with the QTAIM description of bond path topologies that suggest many-fold interactions between the host and the guest in each of the endohedral systems. Figs S6–S8 provide detailed insight into the nature of these interactions evaluated using the X-ray crystal geometries of the H_2_O@C_70_ and H_2_O**···**HF@C_70_ systems without and with the presence of the octaethylporphyrinato-nickel(II) moiety.

The C_70_ molecule is represented as an ellipsoid in 3D; the equilibrium position of the H_2_O**···**HX dimers was near at the center of the cage, oriented along the major axis. This positioning of the dimer is observed experimentally for H_2_O**···**HF@C_70_^16^; our calculations for this and for the H_2_O**···**HCl and H_2_O**···**HBr dimers inside the cage concurs with the experimental observation. However, our geometry optimization of the endohedral system with the dimer along the minor axis forced the dimer to rotate back to a configuration illustrated in Fig. 1, which must be due to the significant steric constraints of the cage. The results of QTAIM, RDG-NCI and IGM analyses are in good agreement with this view. It is quite unambiguous from Figs 4–6 that the dimer in its alignment along the major axis maximizes intermolecular interactions between it and interior of the host. The results of our binding energy calculation (assuming the entire guest and the C_70_ as two monomers of each endohedral system) are in line with this (see Text S2). This also explains why the positional exchange of the H_2_O and HF was not detected experimentally at room temperature^16^. The ^13^C NMR spectra of H_2_O**···**HF@C_70_ displayed nine signals due to its C_5v_ symmetry; this is in contrast with what could be expected for the averaged D_5h_ symmetry of both H_2_O@C_70_ and HF@C_70_ if dynamic motion of the encapsulated species occurred^16^.

### Effect of encapsulation on the frontier orbital energies, ionization potential and electron affinity

We calculated the Kohn-Sham HOMO-LUMO gap for C_70_ to be 1.71 eV, in excellent agreement with the DFT-Generalized Gradient Approximation value of 1.714 eV reported previously^58^. From the Tauc plot, the optical energy gap of the C_70_ thin film was reported to be 1.66 eV, exhibiting a semiconductor-like behavior in optical absorption properties, notwithstanding its molecular character^59–62^. The encapsulation of H_2_O**···**HF, H_2_O**···**HCl, and H_2_O**···**HBr inside the C_70_ cage reduces the HOMO-LUMO gap, with values of 1.69, 1.65, and 1.64 eV, respectively; the result is qualitatively in agreement with that obtained using the well-known functional B3LYP (Table S3). This change is predominantly due to the energy of the LUMO level that becomes more negative^63^. This is not unexpected since the C‒C bonds of the C_70_ cage are affected by various H**···**C, O**···**C and C**···**X (X = F, Cl, Br) intermolecular interactions with the guest species. This geometric modification increases the overlap between molecular states and reduces the gap between the frontier orbitals. The decrease of the HOMO-LUMO gap due to encapsulation is consistent with previous findings^28,58,64^. It is also consistent with the marginal decrease in the vertical ionization potential and fundamental and optical gaps (lowest singlet excitation energies), and a negligible increase in the vertical electron affinity of the encaged C_70_ systems compared to isolated C_70_ (Table S4).

**Figure 7:**
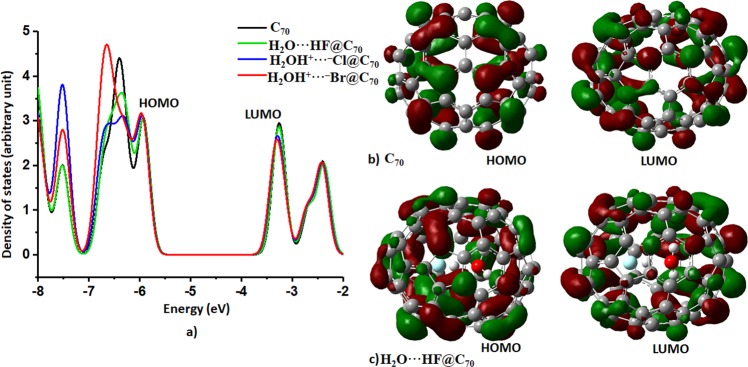
(**a**) Total density of states spectra of isolated and complexed C_70_, (**b**) the nature of localization of HOMO and LUMO (0.02 a.u.) in isolated C_70_ and (**c**) the nature of localization of HOMO and LUMO (0.02 a.u.) in H_2_O···HF@C_70_ (0.02 a.u.), in which, the latter justifies that the guest species contributes nothing to the development of the frontier orbitals.

Figure 7 illustrates the frontier orbitals of C_70_ and H_2_O···HF@C_70_. Both the HOMO and LUMO are localized on the skeletal framework of isolated C_70_. Encapsulation of H_2_O···HF slightly shifts the energy levels of HOMO and LUMO, reducing the energy gap between them. However, this does not lead to a change in the nature of both these frontier orbitals, as evidenced in the density of states (DOS) spectra (Fig. 7a). In other words, our analysis of the atom-projected DOS spectra suggests that the encapsulated species does not contribute to the frontier orbitals (see Fig. 7c for H_2_O**···**HF@C_70_, as example). This is also the case for the other close-lying orbitals (HOMO–1, HOMO–2, LUMO + 1 and LUMO + 2, etc.) of the complexes; they are completely derived from orbitals states of the C_70_ only, and hence the charge transfers between the cage and the guest species is negligibly small. This might explain why the experimentally observed electronic transition properties of the H_2_O**···**HF@C_70_ and C_70_ are very similar, with only marginal changes, compared to isolated C_70_^16^. That the guest species does not contribute to the frontier orbitals is analogous with the organic-inorganic methylammonium lead triiodide (MAPbI_3_) semiconductor. In this, the organic cation MA, entrapped inside the inorganic cage, does not contribute either to the conduction band minimum (CBM) or to the valence band maximum (VBM) that are built from significant contributions from s and p orbitals of the Pb and I atoms. Yet the cation plays a vital role in providing geometrical stability to the MAPbI_3_ system, as well in reducing the bandgap of the material compared to PbI_2_ (1.6 *vs* 2.3 eV).

### Effect of encapsulation on vibrational frequencies and intensities

The elongation of the HF, HCl and HBr bond distances upon the formation of isolated H_2_O···HF, H_2_O**···**HCl and H_2_O**···**HBr complexes (Fig. 1) are accompanied by a significant red-shift in their fundamental vibrational frequencies and a concomitant increase in the intensities of the corresponding IR bands. The red-shift of the HX bond stretching frequency is 457, 506 and 476 cm^−1^ for HF, HCl and HBr, respectively. The increase in the intensity of the corresponding bands is approximately 11, 42 and 217 times larger than the corresponding values of 75, 31 and 5 km mol^−1^ for the isolated HX molecules, respectively.

**Figure 8:**
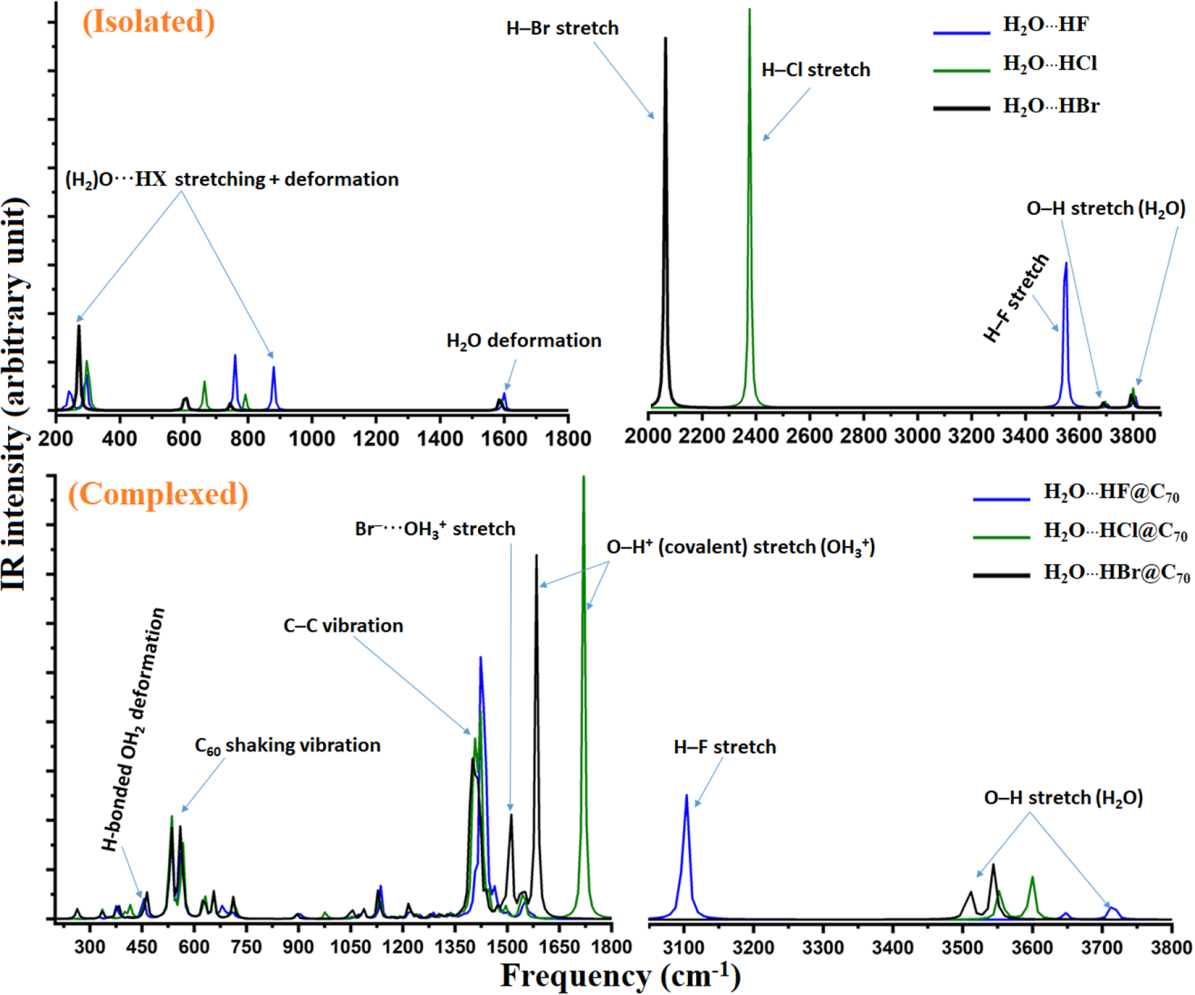
Simulated IR spectra of (**a**) isolated H_2_O···HF, H_2_O···HCl and H_2_O···HBr dimers (top), and that of (**b**) H_2_O···HF@C_70_, H_2_OH^+^···^−^Cl@C_70_ and H_2_OH^+^···^−^Br@C_70_ (bottom). Important vibrational modes are marked by arrows.

Encapsulation of H_2_O**···**HCl and H_2_O**···**HBr inside the C_70_ cages further increases the HCl and HBr bond lengths because of proton transfer (*vide supra*). As is seen in the simulated IR spectra shown in Fig. 8, there is no HX (X = Cl, Br) normal mode vibration in the spectra. Rather, we observe an (H_2_)O–H^+^ stretching vibration for these two endohedral systems that occur at frequencies of 1720 and 1584 cm^−1^, respectively, with the intensities of the corresponding IR bands of 134.6 and 106.8 km mol^−1^, respectively; these are indeed measurable.

For H_2_O**···**HF@C_60_, however, the proton transfer is not as facile. Whereas the HF stretching vibration is centered at 3102 cm^−1^, the intensity of the corresponding IR band is 45.3 km mol^−1^. Compared to the intensities of the isolated HF molecule (75 km mol^−1^) and the isolated H_2_O**···**HF dimer (810 km mol^−1^), the intensity of the IR band in H_2_O**···**HF@C_60_ is appreciably diminished; this does not explain the apparent correlation between intensity reduction and elongation of the H–F bond. The redshift of the band compared to the isolated H_2_O**···**HF and HF systems is 446 and 903.6 cm^−1^, respectively.

The H_2_O species in these three endohedral systems offer very interesting IR characteristics. The O–H symmetric and asymmetric vibrations of the H_2_O coordinated to HX species without the cage (i.e., in the isolated dimers) occur between 3690 and 3805 cm^−1^. Within the cage, the frequencies of these vibrations are red-shifted and occur at 3500 and 3720 cm^−1^ (Fig. 8). According to the literature^65^, the intensities of the corresponding vibrational modes should increase. However, we found them to decrease significantly. For instance, the respective intensities of these two IR bands for isolated H_2_O**···**HF were 11.6 and 58.5 km mol^−1^, but 2.0 and 6.5 km mol^−1^ for H_2_O**···**HF@C_70._ For H_2_O**···**HCl, they were respectively 19.3 and 62.0 km mol^−1^, but 9.8 and 13.7 km mol^−1^ for H_2_OH^+^**···**^−^Cl@C_70_. Similarly, for H_2_O···HBr and H_2_OH^+^**···**^−^Br@C_70_, the intensities decreased from 21.0 and 58.9 km mol^−1^ to 11.9 and 16.5 km mol^−1^.

Because of this apparent anomaly in the intensity profile, analytical second derivative calculations were performed on the endohedral geometries of all the three guest dimers by removing the C_70_ cage. Positive frequencies were found in all cases, indicative of these being in meta-stable states. The simulated IR spectra are shown in Fig. S9. Although the frequency centers in the simulated spectra were somewhat shifted because of the removal of the cage, the intensities of most of the vibrational bands were found to be markedly larger than those shown in Fig. 8. For instance, the HF stretching frequency (intensity) in [H_2_O···HF] occurs at 3197 cm^−1^ (1273 km mol^−1^), whereas that of the O–H^+^ band occurs at 1894 cm^−1^ (3175.1 km mol^−1^) and 1754 cm^−1^ (2992 km mol^−1^) for [H_2_OH^+^···^−^Cl] and [H_2_OH^+^···^−^Br], respectively (Fig. S9). This provides unequivocal proof that the reduction in the band intensity is a consequence of the profound electrostatic shielding effect of the C_70_ cage. It explains why in many previous experimental studies the stretching and bending vibrational bands of HF and H_2_O, as well as those associated with the intermolecular interactions of the encaged species^6^, were not detected. Specifically, the IR bands associated with the HO–H and F–H bonds and those between H_2_O and the cage in H_2_O···HF@C_70_^16^, as well as the corresponding vibrational bands of H_2_O in H_2_O@C_60_^6^ and in H_2_O@C_70_^11^, could not be identified in experimental studies.

Common in both the isolated and complexed C_70_ systems are the normal modes of the C‒C stretching vibrations, which have often been identified experimentally and used to demonstrate that the encaged species residing near the center of the cage is inert^6,7,11,16^. Our calculations suggest that there are three such reasonably strong vibrations in isolated C_70_. Two of them are nearly degenerate and appear at a frequency (intensity) of 1436 cm^−1^ (81 km mol^−1^), and the other at 1470 cm^−1^ (9.8 km mol^−1^). These are in excellent agreement with those of the experimentally measured values of 1430 and 1459 cm^−1^, with the first weaker in intensity^16^. The first mode for H_2_O···HF@C_70_ was reasonably strong and was observed at 1427 cm^−1^; the latter was only observed as a shoulder (no value reported)^16^. In the simulated complex geometries (Fig. 7), these modes showed up at frequencies (intensities) of 1436 and 1462 cm^−1^ (34.0 and 8.4 km mol^−1^) for H_2_O···HF@C_70_, respectively; evidently, DFT predicts these spectra very accurately. The corresponding values for H_2_OH^+^···^−^Cl@C_70_ were 1425 and 1447 cm^−1^ (27 and 6.4 km mol^−1^), and 1419 and 1440 cm^−1^ (26.4 and 2.0 km mol^−1^) for H_2_OH^+^···^−^Br@C_70_. Because of only a marginal change in the C‒C vibrational spectra between the isolated and complexed C_70_, it was misleadingly concluded that the guest species is inert inside the cage for H_2_O···HF@C_70_ – a conclusion that was drawn for several other such endohedral systems as well, including H_2_O@C_60_^6,7,11^.

### Effect of encapsulation on the volume of C_70_

The centroid-to-centroid distance between the two five-membered rings of the C_70_ cage placed at the opposite extremes of the major axis (Fig. 1) is 7.964, 8.007 and 8.205 Å for H_2_O**···**HF@C_70_, H_2_OH^+^**···**^−^Cl@C_70_ and H_2_OH^+^**···**^−^Br@C_70_, respectively; for the isolated C_70_ molecule it is 7.945 Å. This shows that there is a marginal elongation of the major axis of the cage due to the enclosed dimers. This is accompanied by a contraction along the minor axis so that overall the volume of the cage decreases by 101.43, 128.98 and 138.89 a.u. for H_2_O**···**HF@C_70_, H_2_OH^+^**···**^−^Cl@C_70_ and H_2_OH^+^**···**^−^Br@C_70_, respectively, compared to the free C_70_ volume of 5053.83 a.u.

The 0.001 a.u. isodensity envelope suggested by Bader and co-workers^66^ was used for the calculation of atomic volume (Table S5–S6). When the 0.002 a.u. isodensity envelope was used, the corresponding decrease of the volume of the endohedral C_70_ relative to a volume of 4435.30 a.u. for free C_70_ was 41.56, 66.56 and 76.59 a.u., respectively. Irrespective of the nature of the chemical interaction between the encaged species and the cage, the volume of H_2_O**···**HX(X = F, Cl, Br), compared to the volume of the isolated species, is reduced on encapsulation. Our calculations show that the reduction in the (0.001 a.u. isodensity envelope) volume of the guest species is 51.32, 159.96 and 188.49 a.u. for H_2_O**···**HF@C_70_, H_2_OH^+^**···**^−^Cl@C_70_ and H_2_OH^+^**···**^−^Br@C_70_, respectively; these are relative to volumes of 302.68, 453.63 and 498.62 a.u. for isolated H_2_O**···**HF, H_2_O**···**HCl and H_2_O**···**HBr, respectively. This is consonant with an interpretation provided previously for H_2_O**···**HF@C_70_^16^, in which, the study did not involve any real determination of the volume of either the free or encaged C_70_; yet it was speculated that the contraction of the O**···**H bond in H_2_O**···**HF is a result of compression of the H_2_O and HF inside the limited space of the cage environment.

### Effect of encapsulation on the polarizability and dielectric properties of C_70_

Polarizability *α* is another landmark property of fullerene chemistry since fullerenes are highly polarizable molecules^67^. It is calculated to be 637.51 a.u. for free C_70_. Encapsulation of H_2_O**···**HF, H_2_O**···**HCl and H_2_O**···**HBr marginally increases α by 2.92, 6.12 and 7.31 a.u., respectively, which describes the change (Δ*α*(guest@C_70_) = *α*(guest@C_70_) − *α*(C_70_)) in the overall polarizability upon the addition of the dimer. Considering the additive scheme, we found that the deviation Δ*α* (Δ*α*(guest@C_70_) = *α*(guest@C_70_) − (*α*(guest) + *α*(C_70_))) to be negative: −7.32 a.u. for H_2_O**···**HF@C_70_, −12.24 a.u. for H_2_OH^+^···^−^Cl@C_70_ and −16.16 a.u. for H_2_OH^+^**···**^−^Br@C_70_. This suggests a decrease in polarizability (i.e., compression of electron clouds), analogous to what was observed for noble gas endofullerenes with C_36_,C_50_, and C_60_^68^. It is a result of the prevailing pressure of carbon cage that compresses the guest, resulting in a decrease in the total polarizability^69^.

The change *α* affects the dielectric constant *ε* of C_70_, a property that is often used to characterize the optical efficiency of semiconducting materials. Within the framework of the well-known Clausius-Mossotti model^70,71^, *ε* can be expressed as: $\varepsilon =1+\tfrac{8\pi \alpha }{3v}/1-\tfrac{4\pi \alpha }{3v}$, where *α* and *v* are the static dipolar polarizability and the volume of the molecular species, respectively. Based on this model, our calculation gave *ε* values of 4.36 (3.94, experimental), 4.19, 4.20 and 4.21 for C_70_, H_2_O**···**HF@C_70_, H_2_OH^+^**···**^−^Cl@C_70_ and H_2_OH^+^**···**^−^Br@C_70_, respectively (Table S7). This suggests a marginal decrease in the dielectric constant of the encapsulated species. Previous studies demonstrate that provided the dipole cannot reorient fast enough in composites, this causes the dielectric constant to decrease^72^. Others propose that the low dielectric constant of organic photovoltaics assists the exciton to present at larger distances^73^.

## Conclusion

Using DFT and first-principles investigations we have demonstrated the reactivity of three fundamentally important complex systems, H_2_O**···**HX (X = F, Cl, Br), inside the fullerene C_70_ cage. The geometries and vibrational characteristics of the H_2_O**···**HF system obtained at the PBE level of theory adequately explain the experimentally-reported features. The PBE stabilization energies for the dimers were shown to be comparable with those evaluated with CCSD(T). At the same time, we have demonstrated using geometrical, electron density and vibrational characteristics that the C_70_ cage interior serves as a super-catalyst for HCl and HBr bond dissociation, enhancing the acidity of these acids by bond cleavage and assisting in complete proton transfers to H_2_O. This leads to the formation of OH_3_^+^X^−^ (X = Br, Cl) ion pair complexes within the cage. This was not the case for the H_2_O**···**HF dimer inside C_70_, however. This is certainly the result of the significant basicity of F^−^ compared to Cl^−^ and Br^−^.

We summarize our observations as follows.

1. The C_70_ cage interior is not hydrophobic and the guest species are not inert, as often contended^6,7,11,16^. A single water molecule when encaged will experience significant hydrogen bonding; this occurs regardless of whether we are dealing with an isolated water dimer^11^ or an H_2_O**···**HX dimer. This observation is in line with others in which the effective pair interaction is not hydrophobic, yet the solvation properties are; hence fullerenes serve as an example in which hydrophobic interaction cannot be deducted from hydrophobic solvation^74^.
2. The C_70_ cage interior has a tendency to donate charge density to the guest species.
3. Because the C_70_ cage interior is polarizable, its effect plays a vital role that is largely responsible for the development of many anomalous features that were undetectable experimentally in previous studies (e.g., IR characteristics of intermolecular interactions).
4. Because of its nature as stated in 2 and 3, C_70_ prefers to serve as a cationic host upon encapsulation of two guest molecules.
5. It has the potential to provide a terrace to the guest species for facilitating efficient proton transfer reactions between them.
6. The C_70_ cage interior has the ability to screen the electrostatic (dipolar) field of the guest species, hence limiting the observability of many vibrational bands that are IR active. This explains why experiments often fail to show the IR spectra of the entrapped species inside the host.
7. Charge rearrangement, bond polarization and ion-pair formation are likely consequences of an accommodation of a host species, especially for dimer molecules.

The C_70_ cage interior provides an elegant and innovative terrace to electrogenerate reactive species between H_2_O and HX. This is fully consistent with our similar investigations for other species (such as F_2_ and mixed dihalogen derivatives), as well as other systems such as NH_3_ and HX (X = F, Br, Cl), in the presence of H_2_O, that merit investigation to delineate the novelty of the catalytic profile of the C_70_ cage interior. In fact, our preliminary results, to be reported elsewhere, show that F_2_ inside the C_70_ cage can be completely dissociated into two F^−^ ions in presence of a single molecule of H_2_O. The mechanistic details involved in this and other similar systems will surely uncover the novel physical chemistry and catalytic detail of these materials.

## Materials and Methods

The Gaussview 05 package^75^ was used for generating the geometries of isolated molecules (HF, HCl, HBr, H_2_O and C_70_), and their dimers (H_2_O**···**HX (X = F, Cl, Br) and (H_2_O**···**HX@C_70_ (X = F, Cl, Br)). The endohedral complexes (H_2_O**···**HX@C_70_ (X = F, Cl, Br)) were generated using the same procedure. The Cartesian coordinates of these systems were used for energy-minimizations using the Gaussian 09^26^ package. For reasons discussed in the Results and Discussion section, the PBE functional implanted in Gaussian 09, together with the 6-311G(d,p) basis set, was used. The reliability of the functional in obtaining the experimental geometry and vibrational spectra of the H_2_O**···**HF@C_70_ system is discussed in both Fig. S1 and the Results and Discussion section. The same method was used for calculations to obtain the eigenvalues of the Hessian second derivative matrix; in all cases, positive eigenvalues were obtained. This confirmed that the various monomer, dimer and endohedral geometries discussed in this study are all local minima. Tight convergence and ultrafine integration grids were used.

The potential energy surface (PES) rigid scans were performed on the isolated and C_70_ complexed H_2_O**···**HCl (and H_2_O**···**HCl) geometries with MP2/aug-cc-pVTZ to show the effect of C_70_ cage on the nature of proton transfer from the HCl (and HBr) species to the H_2_O molecule. The PBE energy-minimized geometries were used.

The binding energies associated with the H_2_O**···**HX dimers were evaluated at various levels of theory (PBE, MP2 and CCSD(T)) in conjunction with the aug-cc-pVTZ basis set; the PBE/6-311G(d,p) optimized geometries were used. To demonstrate the qualitative (and quantitative) reliability of the results of the PBE function, other functionals such as M06-2X, M06-2X-D3, ωB97XD, B97D3, PBE-D3, and PBE0-D3 (as implemented in Gaussian 09) were invoked to estimate the energies of the H_2_O**···**HF dimer and compared. Similar calculations for H_2_O**···**HF@C_70_, H_2_OH^+^···^−^Cl@C_70_ and H_3_OH^+^···^−^Br@C_70_ were performed with B97D3 to estimate the binding energy between the host and the guest species.

The charge density (*ρ*_b_), the Laplacian of the charge density (∇^2^*ρ*_b_) and the total energy density (*H*_*b*_) at bond critical points and the delocalization indices between various atom-atom pairs were evaluated within the framework of the Quantum Theory of Atoms in Molecules (QTAIM)^50^ using the AIMAll package^51^. The atomic volumes *v*_*i*_ are calculated within the framework of this theory, which were used to calculate the total volume of each of the monomers, dimers and endohedral systems using $\nu =\sum _{i=1}^{N}{\nu }_{i}$, where the sum is over all atoms in a given molecule. The mean static dipolar polarizability *α* of each of these systems was calculated using $\alpha =\tfrac{1}{3}({\alpha }_{xx}+{\alpha }_{yy}+{\alpha }_{zz})$, where the *α* values are the three principal (diagonal) components of the 3 × 3 matrix given below.

$$(\begin{array}{lll}{\alpha }_{xx} &  & \\  & {\alpha }_{yy} & \\  &  & {\alpha }_{zz}\end{array})$$

The Independent Model (IGM)^55^ and Reduced Density Gradient Noncovalent Interaction (RDG-NCI)^54^ analyses were performed to examine the nature of intermolecular interactions in the isolated dimers and between it and the cage interior. Depending on the evaluation of specific electron density properties and visualization, software packages such as Multiwfn^76^, AIMAll^51^ and VMD^77^, as well as in-house codes, were used. The HOMO, LUMO and the Kohn-Sham gap (LUMO-HOMO) energies for the isolated and complexed C_70_ systems obtained with B3LYP/6-31G* were compared with those evaluated with PBE/6-311G**; the geometries obtained for these systems at the latter level of theory was used. Further calculations were performed at these levels of theory to examine the nature of the total and atom-projected density of states spectra, vital for a fundamental understanding of the role the guest species plays in building the frontier orbitals. The Gausssum 03 and Origin 2018^78,79^ packages were used for this purpose.

The geometries of the H_2_O**···**HF, H_2_O**···**HCl and H_2_O**···**HBr isolated dimers were also optimized in the first excited state using time-dependent density functional theory (TD-DFT) with PBE/6-311G(d,p). Similar calculations were performed for these systems by providing an additional electron. The goal for these calculations was to examine the extent of proton transfer feature in the excited and anionic states, and to compare this with that revealed when these dimers were encaged inside the C_70_ in their electronic ground states.

## Supplementary information

manuscript
