# Supplementary material for: C70 Fullerene Cage as a Novel Catalyst for Efficient Proton Transfer Reactions between Small Molecules: A Theoretical study
Source: Sci Rep. 2019 Jul 23;9:10650. doi: 10.1038/s41598-019-46725-4 (PMC6650427; doi:10.1038/s41598-019-46725-4)
Supplement: Supplementary file 1 — manuscript [file 41598_2019_46725_MOESM1_ESM.pdf]

C_{70} Fullerene Cage as a Novel Catalyst for Efficient Proton Transfer Reactions between Small Molecules: A Theoretical Study

Pradeep R. Varadwaj,[a,b]* Arpita Varadwaj,[a,b] Helder M. Marques[c]

[a] Department of Chemical System Engineering, School of Engineering, The University of Tokyo 7-3-1, Hongo, Bunkyo-ku, Japan 113-8656

[b] The National Institute of Advanced Industrial Science and Technology (AIST), Tsukuba, Ibaraki 305-8560, Japan

[c]Molecular Sciences Institute, School of Chemistry, University of the Witwatersrand, Johannesburg, 2050, South Africa

Supplementary information

Table S1. Selected Physical Properties of Isolated $H_2O\cdots HF$ without and with C_{70}. [a,b]

System	Method[c]	Δ*E*[c]	Δ*E*(BSSE)[c]	*r*(O···H)	*r*(H-F)	*r*(O···F)	∠O···H-F	∠HOH	*r*(O-H(H₂O))	Δ[d]
$H_2O\cdots HF$	PBE/6-311G**	-12.86	-8.30	1.685	0.950	2.623	168.5	104.3	0.971	0.735
	PBE/aug-cc-pVTZ	-9.43	-9.32							
	MP2/aug-cc-pVTZ	-8.98	-8.12							
	CCSD(T)/aug-cc-pVTZ	-8.87	-8.01							
$H_2O\cdots HF@C_{70}$	PBE/6-311G**	-13.33	-9.30	1.485	0.973	2.457	175.4	104.7	0.975	0.511
	PBE/aug-cc-pVTZ	-9.53	-9.44							
	MP2/aug-cc-pVTZ	-9.20	-8.56							
	CCSD(T)/aug-cc-pVTZ	-9.23	-8.57							

[a] Geometries of $H_2O\cdots HF@\mathbf{C_{70}}$ and $H_2O\cdots HF$ were obtained with PBE/6-311G**.

[b] Binding energies Δ*E* and Δ*E*(BSSE) in kcal mol^{-1}, bond distances in Å, and bond distances in deg. Δ*E*($H_2O\cdots HF$) = *E*($H_2O\cdots HF$) – *E*(H_2O) – *E*(HF); Δ*E*(BSSE) = Δ*E* + *E*(BSSE), where *E* is the total electronic energy of the individual species, and *E*(BSSE) is basis set superposition error energy.

[c] PBE, MP2 and CCSD(T), in conjunction with the aug-cc-pVTZ basis set, energies were obtained on the PBE/6-311G** energy-minimized geometries of $H_2O\cdots HF@\mathbf{C_{70}}$ and $H_2O\cdots HF$.

[d] Δ is the proton coordinate distances difference (Å),) *r*(H··O) – *r*(H–F)

Table S2. Selected Physical Properties of the $H_2O\cdots HX$ (X = Cl, Br)with and without C_{70} [a,b,c]

System	Method[c]	ΔE	ΔE(BSSE)	r(O···H)	r(H–X)	r(O···X)	∠O···H–X	∠HOH	r(O–H(H₂O))	Δ [f]
$H_2OH^+\cdots{}^-Cl@C_{70}$ [d]	PBE/6-311G**	-187.02	-186.09	1.405	1.283	2.689	172.925	105.3	0.980	-0.122
	PBE/aug-cc-pVTZ	-187.76	-187.58							
	MP2/aug-cc-pVTZ	-188.62	-186.35							
	CCSD(T)/aug-cc-pVTZ	-188.31	-186.02							
$H_2O\cdots HCl$ [e]	PBE/6-311G**	-11.62	-8.40	1.713	1.337	3.048	175.9	104.5	0.972	+0.376
	PBE/aug-cc-pVTZ	-6.85	-6.76							
	MP2/aug-cc-pVTZ	-6.57	-5.89							
	CCSD(T)/aug-cc-pVTZ	-5.85	-5.18							
$H_2OH^+\cdots{}^-Br@C_{70}$ [d]	PBE/6-311G**	-171.65	-170.24	1.221	1.563	2.760	164.8	105.3	0.982	-0.342
	PBE/aug-cc-pVTZ	-168.47	-168.23							
	MP2/aug-cc-pVTZ	-169.98	-165.68							
	CCSD(T)/aug-cc-pVTZ	-169.28	-164.94							
$H_2O\cdots HBr$ [e]	PBE/6-311G**	-10.43	-7.09	1.745	1.479	3.221	174.7	104.4	0.972	+0.266
	PBE/aug-cc-pVTZ	5.97	-5.86							
	MP2/aug-cc-pVTZ	-6.2	-5.03							
	CCSD(T)/aug-cc-pVTZ	-4.68	-3.57							

[a] Geometries of $H_2O\cdots HX@C_{70}$ and $H_2O\cdots HX$ were obtained with PBE/6-311G(d,p).

[b] Binding energies ΔE and ΔE(BSSE) in kcal mol^{-1}, bond distances in Å, and bond distances in deg.

[c] PBE, MP2 and CCSD(T), in conjunction with the aug-cc-pVTZ basis set, binding energies were obtained on the PBE/6-311G** energy-minimized geometries.

[d] $\Delta E(H_2OH^+\cdots{}^-X) = E(H_2OH^+\cdots{}^-X) - E(H_2OH^+) - E({}^-X)$ (X = Cl, Br); $\Delta E(BSSE) = \Delta E + E(BSSE)$, where E is the total electronic energy of individual species, and E(BSSE) is the basis set superposition error energy.

[e] $\Delta E(H_2O\cdots HX) = E(H_2O\cdots HX) - E(H_2O) - E(HX)$; $\Delta E(BSSE) = \Delta E + E(BSSE)$, where E is the total electronic energy of individual species, and E(BSSE) is the basis set superposition error energy.

[f] Δ is the proton coordinate distances difference (Å),) $r(H\cdots O) - r(H–X)$

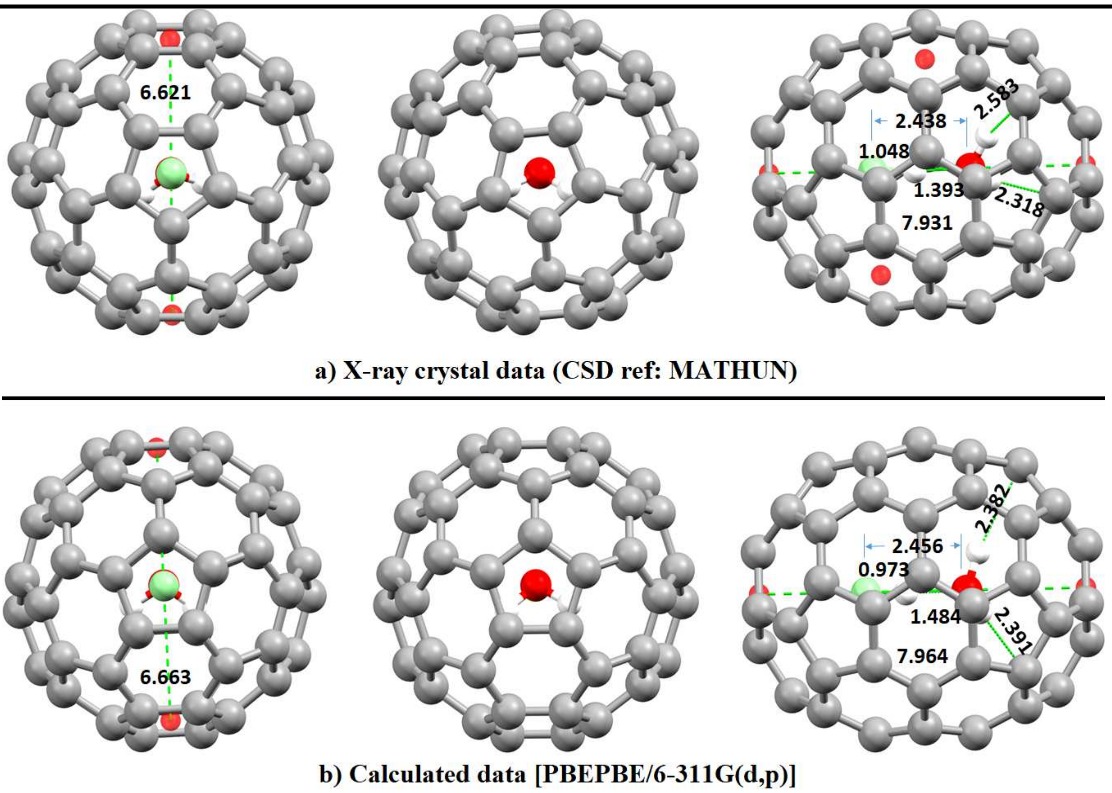

Figure S1. Comparison between various views of experimental and theoretically simulated geometries of $H_2O\cdots HF@C_{70}$. Note that the H atom positions in the X-ray diffraction geometry are manipulated through a least squares fitting analysis. Although these are somehow inaccurate, these compare well with the PBE predicted data. Bond lengths are in Å, in which, the experimental $O\cdots H$, $O\cdots F$ and the $C_5\cdots C_5$ (centrod-centrod) distances (1.393, 2.438 and 7.931 Å) were reproduced within < 0.1 Å. The $\angle O\cdots HF$ for the experimental and calculated geometries were 174.6 and 175.4°, respectively, showing the calculated bond angle is reproduced within 1°.

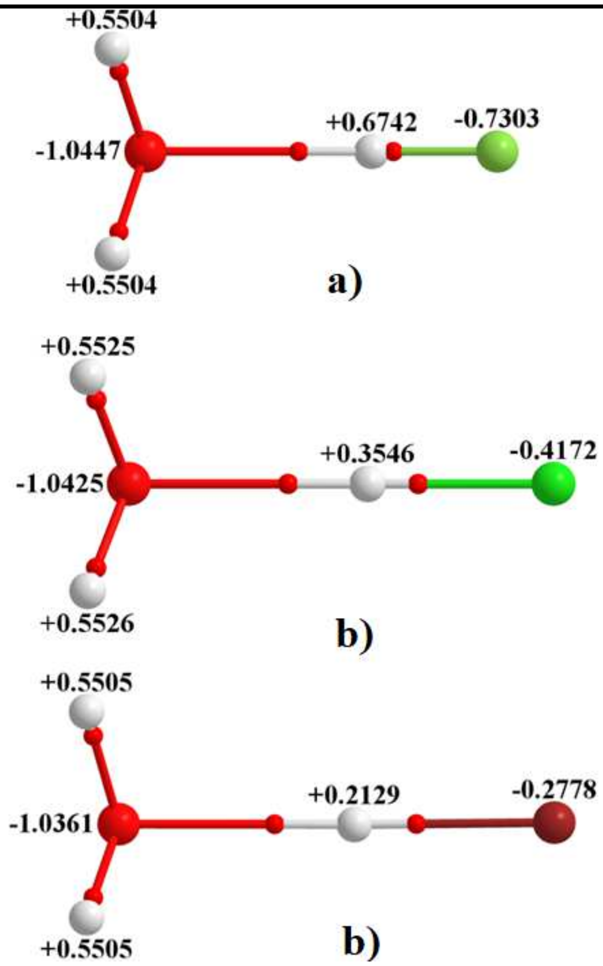

Figure S2. PBE level integrated QTAIM charges (in *e*) for isolated a) $H_2O\cdots HF$, b) $H_2O\cdots HCl$ and c) $H_2O\cdots HBr$.

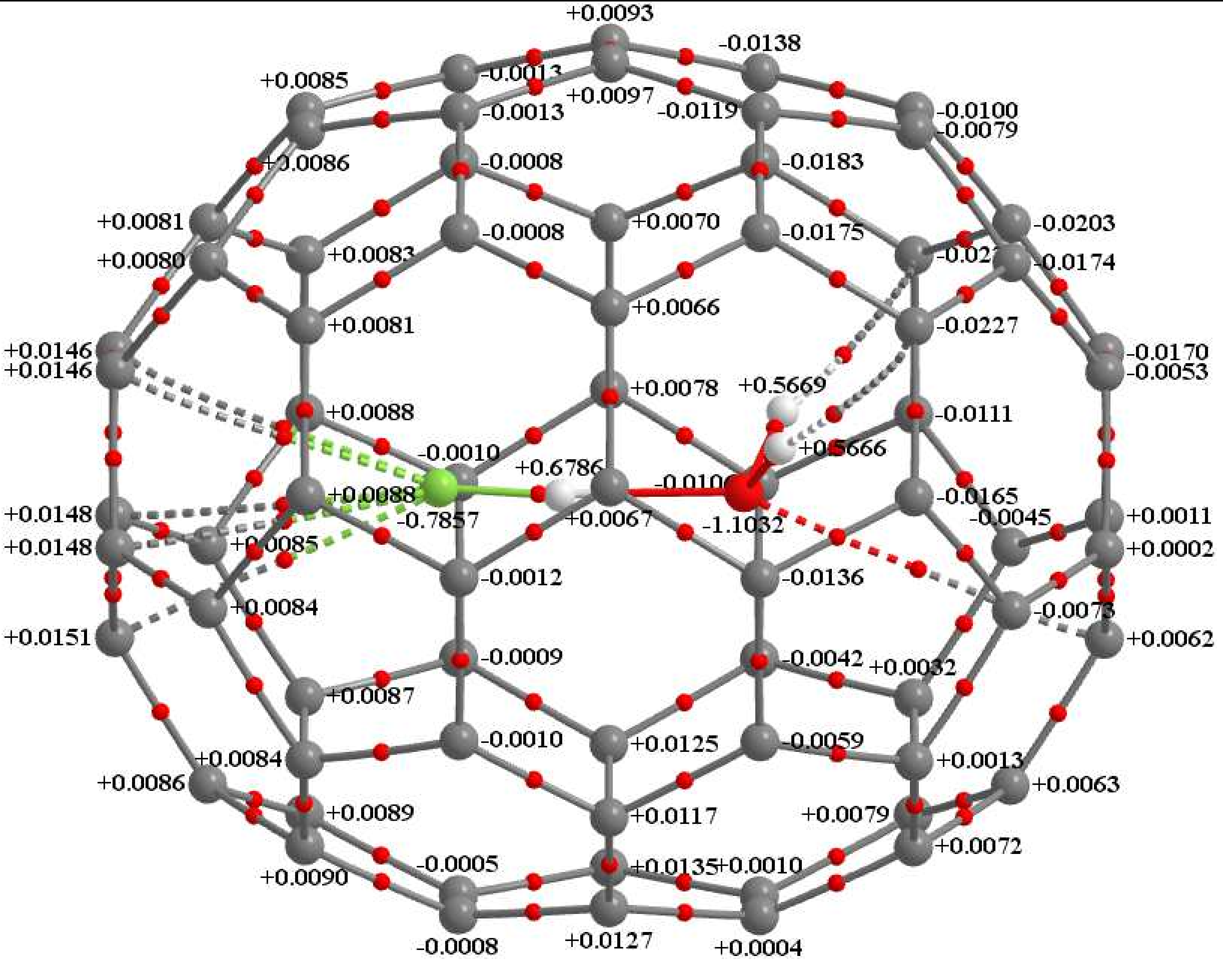

Figure S3. PBE level integrated QTAIM charges (in *e*) for $H_2O\cdots HF@C_{70}$, showing charge redistribution and separation.

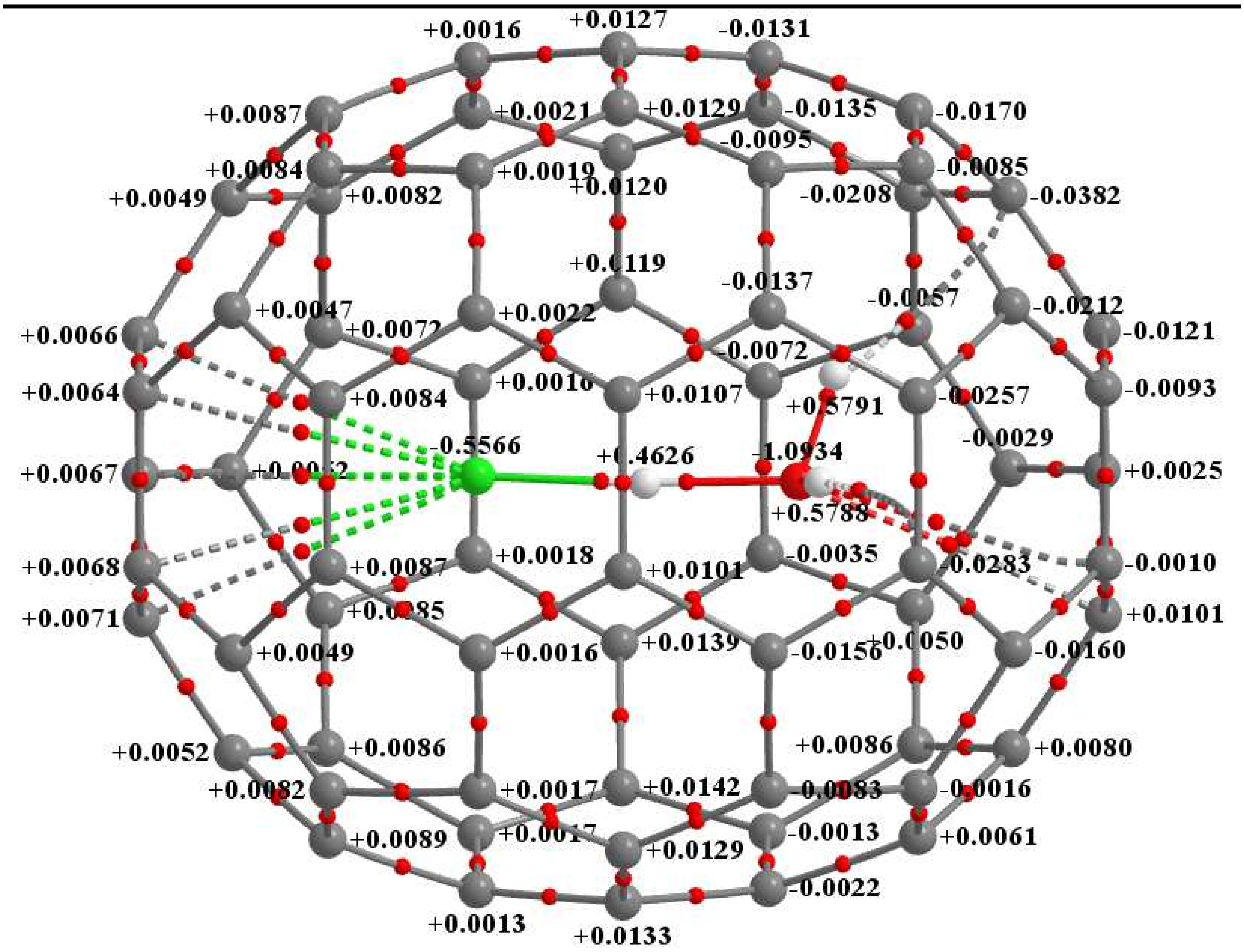

Figure S4. PBE level integrated QTAIM charges (in *e*) for $H_2OH^+\cdots{}^-Cl$, showing charge redistribution and separation.

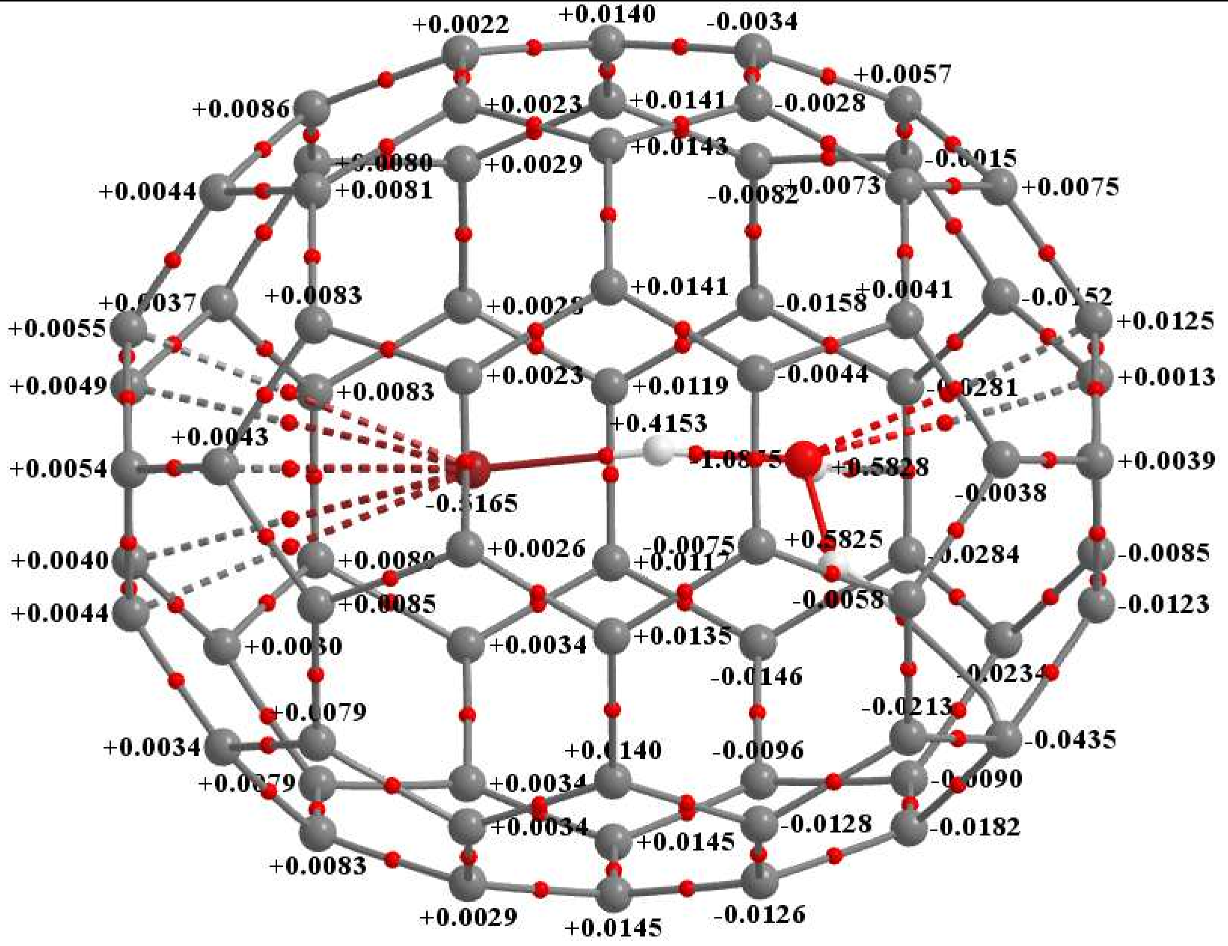

Figure S5. PBE level integrated QTAIM charges (in *e*) for $H_2OH^+\cdots{}^-Br$, showing charge redistribution and separation.

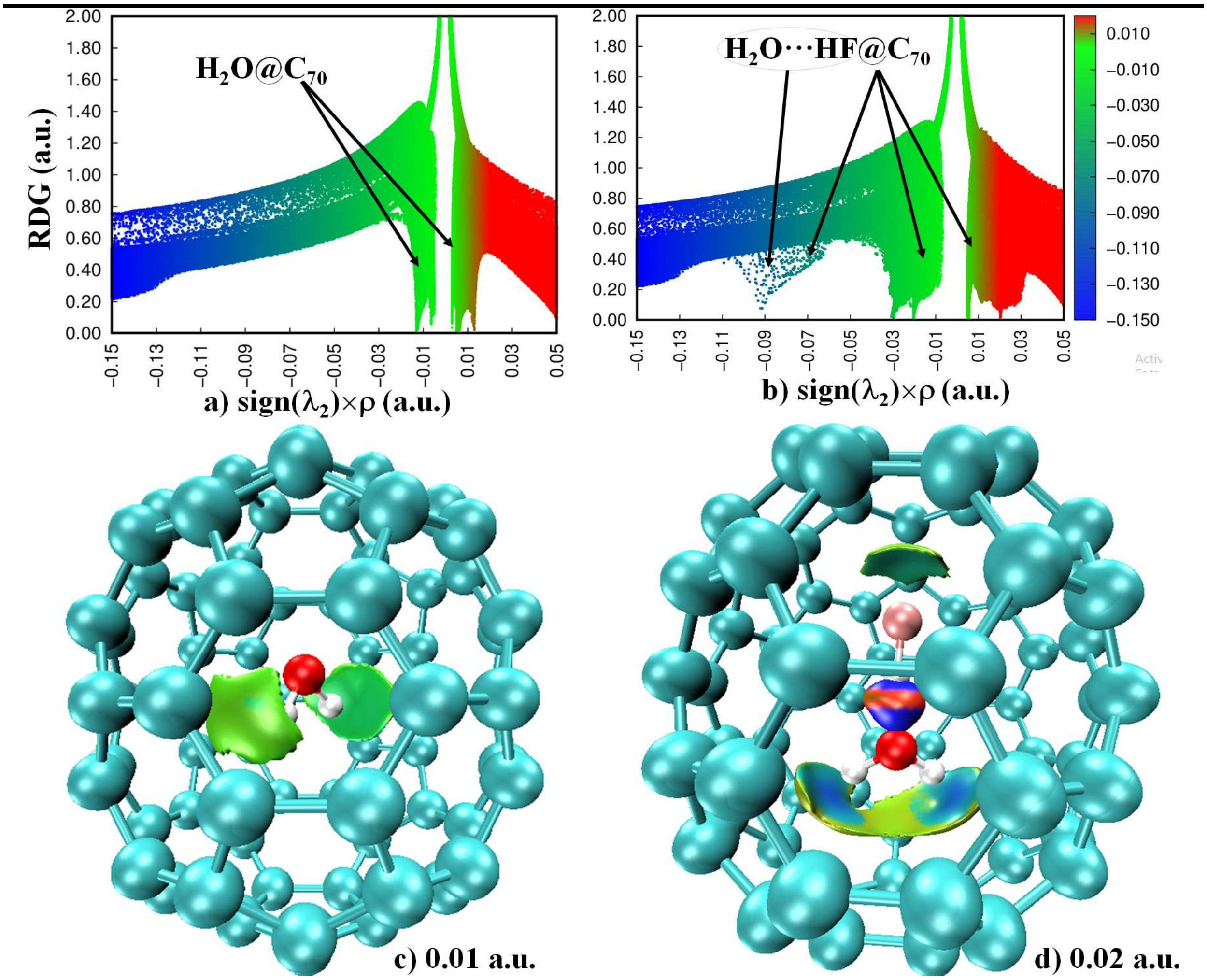

Figure S6. sign(λ_2)×ρ *vs.* RDG 2D plots (0.5 a.u.) for a) $H_2O@C_{70}$, b) $H_2O\cdots HF@C_{70}$. The IGM isosurface plots for the corresponding systems are illustrated in c) 0.01 a.u. and d) 0.02 a.u., respectively. Experimental X-ray crystal geometries of a) $H_2O@C_{70}$ (CSD ref: VAKTIN) b) $H_2O\cdots HF@C_{70}$ (CSD ref: MATHUN) obtained from Cambridge Structure Database, together with promolecular approximation, was used for RDG and IGM analysis. Isosurfaces colored blue, green and red represent reasonably strong, medium-to-weak (attraction) and strongly repulsive interactions, respectively.

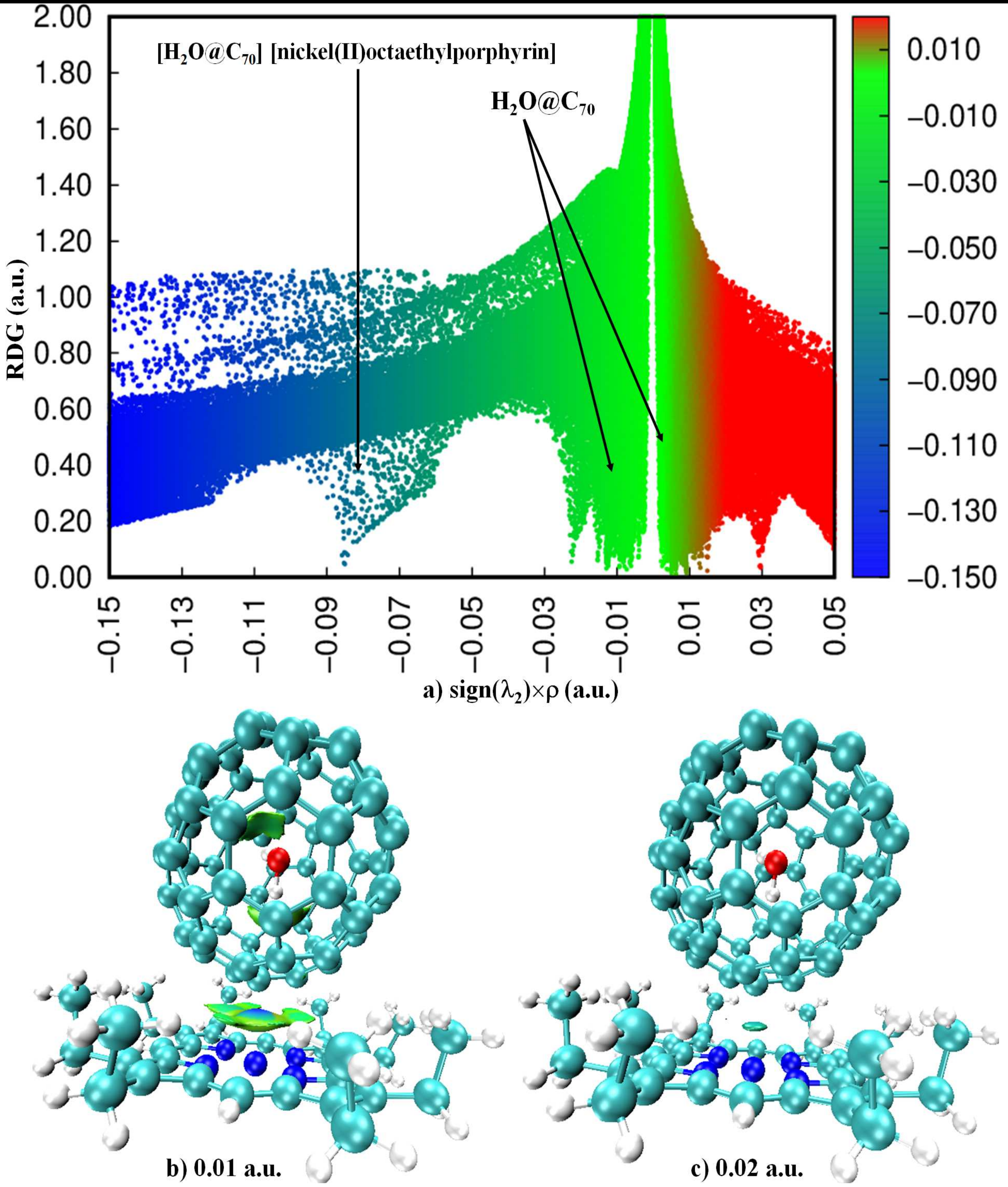

Figure S7. sign(λ_2)×ρ *vs.* RDG 2D plots (0.5 a.u.) for a) $H_2O@C_{70}$ (2,3,7,8,12,13,17,18-octaethylporphyrinato)-nickel(ii)). The IGM isosurface plots for the corresponding system is illustrated in b) 0.01 a.u. and c) 0.02 a.u., respectively. Experimental X-ray crystal geometry of C_{70} Fullerene (2,3,7,8,12,13,17,18-octaethylporphyrinato)-nickel(ii) benzene chloroform solvate hydrate obtained from CSD (CSD ref: VAKTIN), together with promolecular approximation, was used for RDG and IGM analysis. Isosurfaces colored blue, green and red represent reasonably strong, medium-to-weak (attraction) and strongly repulsive interactions, respectively.

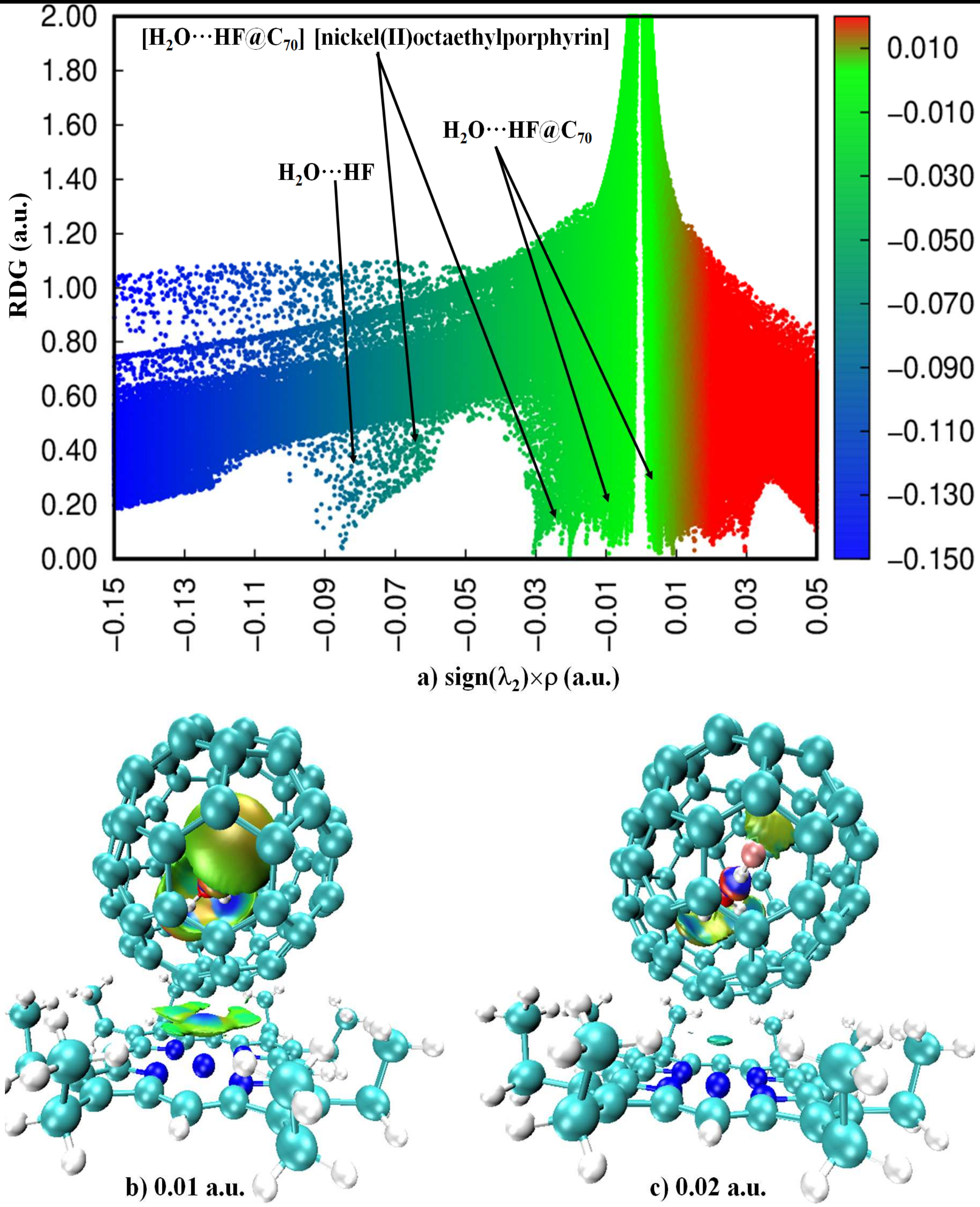

Figure S8. sign(λ_2)×ρ *vs.* RDG 2D plots (0.5 a.u.) for a) $H_2O\cdots HF@C_{70}$ (2,3,7,8,12,13,17,18-octaethylporphyrinato)-nickel(ii)). The IGM isosurface plots for the corresponding system is illustrated in b) 0.01 a.u. and c) 0.02 a.u., respectively. Experimental X-ray crystal geometry of ([2,3,7,8,12,13,17,18-octaethylporphyrinato]-nickel(ii) C_{70} fullerene chloroform hydrogen fluoride benzene solvate monohydrate) obtained from CSD (CSD ref: MATHUN), together with promolecular approximation, was used for RDG and IGM analysis. Isosurfaces colored blue, green and red represent reasonably strong, medium-to-weak (attraction) and strongly repulsive interactions, respectively.

Table S3: Computed energies (in eV) of HOMO, LUMO and Kohn-Sham gap (KSGAP) for isolated and complexed C_{70}, obtained with two different levels of theory. [a]

Species	B3LYP/6-31G*				PBE/6-311G**			
	HOMO	LUMO	KSGAP[b]	Decrease[c]	HOMO	LUMO	KSGAP[b]	Decrease[c]
**Free C_{70}**	-5.91	-3.27	2.65	0.00	-5.87	-4.16	1.71	0.00
**$H_2O\cdots HF@C_{70}$**	-5.90	-3.29	2.61	-0.04	-5.86	-4.18	1.69	-0.02
**$H_2OH^+\cdots{}^-Cl@C_{70}$**	-5.91	-3.33	2.58	-0.07	-5.88	-4.22	1.65	-0.06
**$H_2OH^+\cdots{}^-Br@C_{70}$**	-5.91	-3.34	2.57	-0.07	-5.88	-4.24	1.64	-0.07

[a] PBE/6-311G** geometries for isolated and complexed C_{70} were used.
[b] KSGAP = LUMO - HOMO
[c] Decrease = Complexed (C_{70} with guest) – Free (C_{70})

Table S4: B3LYP/6-31G* **c**omputed energies (in eV) of HOMO, LUMO, Kohn-Sham gap (KSGAP), ionization potential, electron affinity and fundamental gap for isolated and complexed C_{70}, obtained with two different levels of theory. [a]

	HOMO	LUMO	KSGAP	Ionization Potential	Electron Affinity	Fundamental Gap	Optical gap
Free C_{70}	-5.91	-3.27	2.65	7.08	2.11	4.96	2.07
$H_2O\cdots HF@C_{70}$	-5.90	-3.29	2.61	7.04	2.12	4.92	2.05
$H_2OH^+\cdots{}^-Cl@C_{70}$	-5.91	-3.33	2.58	7.07	2.18	4.89	2.02
$H_2OH^+\cdots{}^-Br@C_{70}$	-5.91	-3.34	2.57	7.07	2.19	4.88	2.01

[a] PBE/6-311G** geometries for isolated and complexed C_{70} were used.
[b] KSGAP = LUMO - HOMO
c The optical gaps for the corresponding species were 1.76, 1.73, 1.71 and 1.70 eV, respectively.

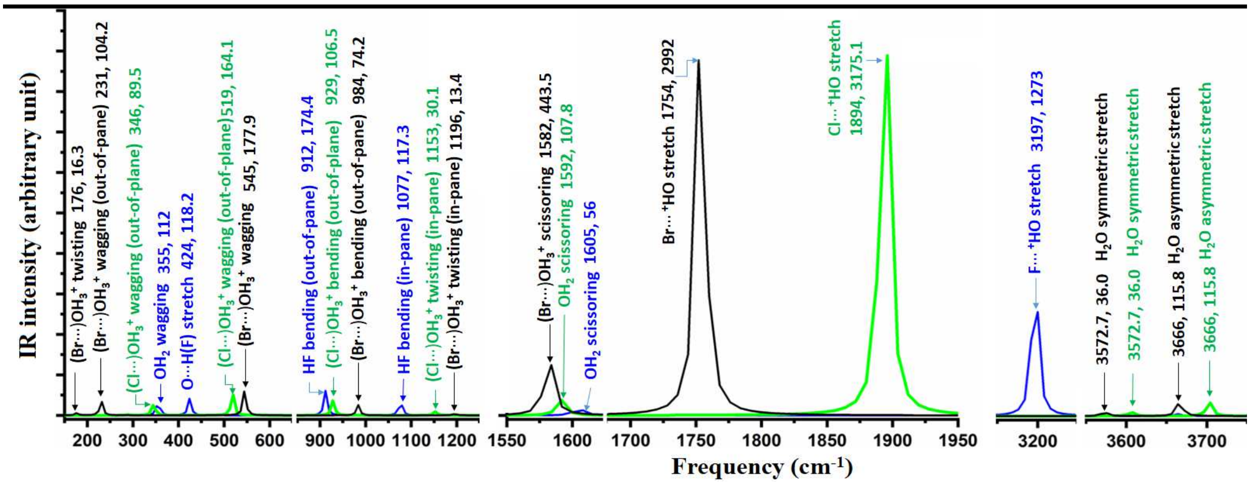

Figure S9. Simulated IR spectra of $H_2O\cdots HF$, $H_2OH^+\cdots{}^-Cl$ and $H_2OH^+\cdots{}^-Br$, obtained on the geometries of these complexes as in C_{70}. The nature of approximate vibration, frequency centers (in cm^{-1}) and intensities ($km\ mol^{-1}$) of the bands are shown for selected modes.

Table S5: PBE level volumes of atomic basins (in a.u.) of isolated C_{70}.

0.001 a.u.		0.002 a.u.	
**Atom**	Volume	Atom	Volume
**C1**	72.56	C1	63.54
**C2**	72.52	C2	63.54
**C3**	72.57	C3	63.54
**C4**	72.53	C4	63.52
**C5**	72.57	C5	63.54
**C6**	72.91	C6	63.65
**C7**	72.05	C7	63.12
**C8**	72.03	C8	63.05
**C9**	72.90	C9	63.62
**C10**	72.04	C10	63.06
**C11**	72.03	C11	63.08
**C12**	72.92	C12	63.63

**C13**	72.05	C13	63.11
**C14**	72.04	C14	63.08
**C15**	72.92	C15	63.65
**C16**	72.07	C16	63.09
**C17**	72.03	C17	63.06
**C18**	72.90	C18	63.64
**C19**	72.04	C19	63.08
**C20**	72.04	C20	63.09
**C21**	72.08	C21	63.34
**C22**	72.08	C22	63.36
**C23**	71.90	C23	63.49
**C24**	71.89	C24	63.52
**C25**	72.04	C25	63.37
**C26**	72.10	C26	63.35
**C27**	71.92	C27	63.49
**C28**	71.99	C28	63.50
**C29**	72.08	C29	63.36
**C30**	72.09	C30	63.36
**C31**	71.89	C31	63.46
**C32**	71.92	C32	63.47
**C33**	72.05	C33	63.38
**C34**	72.04	C34	63.36
**C35**	71.92	C35	63.47
**C36**	71.92	C36	63.53
**C37**	72.08	C37	63.36
**C38**	72.05	C38	63.33
**C39**	71.89	C39	63.48
**C40**	71.89	C40	63.47

**C41**	72.07	C41	63.36
**C42**	72.04	C42	63.34
**C43**	72.01	C43	63.06
**C44**	72.06	C44	63.02
**C45**	72.10	C45	63.35
**C46**	72.08	C46	63.38
**C47**	72.04	C47	63.09
**C48**	72.05	C48	63.07
**C49**	71.99	C49	63.38
**C50**	72.04	C50	63.35
**C51**	72.06	C51	63.06
**C52**	72.05	C52	63.07
**C53**	72.05	C53	63.38
**C54**	72.08	C54	63.35
**C55**	72.03	C55	63.11
**C56**	72.06	C56	63.05
**C57**	72.11	C57	63.36
**C58**	72.03	C58	63.36
**C59**	72.03	C59	63.07
**C60**	72.07	C60	63.10
**C61**	72.95	C61	63.60
**C62**	72.55	C62	63.53
**C63**	72.53	C63	63.56
**C64**	72.89	C64	63.62
**C65**	72.56	C65	63.54
**C66**	72.93	C66	63.65
**C67**	72.87	C67	63.62
**C68**	72.52	C68	63.52

**C69**	72.54	C69	63.58
**C70**	72.89	C70	63.64
Total volume (C_{70})	**5055.83**		**4435.30**

Table S6: PBE level volumes (in a.u.) of atomic basins of C_{70} complexed and free dimers of $H_2O\cdots HX$ (X = F, Cl, Br). The 0.001 a.u. isodensity envelope was used for the evaluation of atomic volumes.

**$H_2O\cdots HF@C_{70}$**		**$H_2OH^+\cdots{}^-Cl@C_{70}$**		**$H_2OH^+\cdots{}^-Br@C_{70}$**	
**Atom**	Volume	Atom	Volume	Atom	Volume
**C1**	70.07	C1	70.20	C1	70.12
**C2**	70.07	C2	70.18	C2	70.12
**C3**	69.99	C3	70.18	C3	70.18
**C4**	69.99	C4	70.10	C4	70.22
**C5**	70.02	C5	70.21	C5	70.21
**C6**	70.80	C6	70.69	C6	70.71
**C7**	70.26	C7	69.93	C7	69.63
**C8**	70.39	C8	69.97	C8	69.72
**C9**	70.91	C9	70.67	C9	70.61
**C10**	70.36	C10	69.86	C10	69.75
**C11**	70.37	C11	69.85	C11	69.75
**C12**	70.96	C12	70.76	C12	70.74
**C13**	70.43	C13	69.92	C13	69.74
**C14**	70.28	C14	69.80	C14	69.66
**C15**	70.88	C15	70.69	C15	70.74
**C16**	70.30	C16	69.93	C16	69.75
**C17**	70.24	C17	69.75	C17	69.67
**C18**	70.80	C18	70.68	C18	70.77

**C19**	70.23	C19	69.82	C19	69.77
**C20**	70.26	C20	69.89	C20	69.81
**C21**	70.98	C21	69.85	C21	69.59
**C22**	71.09	C22	69.91	C22	69.61
**C23**	71.38	C23	70.69	C23	70.21
**C24**	71.33	C24	70.59	C24	70.10
**C25**	71.07	C25	69.91	C25	69.52
**C26**	71.16	C26	70.00	C26	69.72
**C27**	71.40	C27	70.55	C27	70.14
**C28**	71.42	C28	70.62	C28	70.19
**C29**	71.20	C29	69.93	C29	69.53
**C30**	71.11	C30	69.97	C30	69.57
**C31**	71.22	C31	70.56	C31	70.25
**C32**	71.35	C32	70.60	C32	70.15
**C33**	71.16	C33	70.05	C33	69.69
**C34**	71.05	C34	69.98	C34	69.61
**C35**	71.26	C35	70.65	C35	70.28
**C36**	71.20	C36	70.64	C36	70.28
**C37**	71.00	C37	70.00	C37	69.68
**C38**	70.99	C38	69.93	C38	69.63
**C39**	71.13	C39	70.58	C39	70.05
**C40**	71.20	C40	70.49	C40	70.16
**C41**	70.96	C41	71.05	C41	70.94
**C42**	71.06	C42	71.26	C42	71.18
**C43**	70.35	C43	70.32	C43	70.33
**C44**	70.35	C44	70.35	C44	70.30
**C45**	70.67	C45	71.07	C45	70.88
**C46**	70.91	C46	71.09	C46	70.96
**C47**	70.69	C47	70.27	C47	70.15

**C48**	70.66	C48	70.49	C48	70.42
**C49**	70.96	C49	71.07	C49	71.04
**C50**	70.61	C50	70.80	C50	70.69
**C51**	70.29	C51	69.89	C51	69.82
**C52**	70.25	C52	69.76	C52	69.63
**C53**	70.91	C53	70.78	C53	70.82
**C54**	70.90	C54	70.99	C54	70.94
**C55**	70.68	C55	70.57	C55	70.51
**C56**	70.30	C56	70.09	C56	70.07
**C57**	70.63	C57	70.94	C57	70.91
**C58**	70.48	C58	70.85	C58	70.75
**C59**	70.18	C59	70.05	C59	70.13
**C60**	70.58	C60	70.39	C60	70.44
**C61**	71.20	C61	70.89	C61	70.93
**C62**	70.67	C62	70.46	C62	70.49
**C63**	71.88	C63	70.44	C63	70.54
**C64**	71.22	C64	70.49	C64	70.45
**C65**	70.86	C65	70.71	C65	70.73
**C66**	71.35	C66	71.17	C66	71.18
**C67**	71.27	C67	70.87	C67	70.92
**C68**	70.65	C68	70.40	C68	70.44
**C69**	70.68	C69	70.32	C69	70.33
**C70**	70.89	C70	70.42	C70	70.41
**O71**	107.20	O71	91.19	O71	85.38
**H72**	14.27	H72	12.30	H73	12.37
**H73**	14.29	H73	11.87	H74	11.62
**H74**	8.85	H74	14.34	H71	15.75
**F75**	106.74	Cl75	163.97	Br75	185.00
Total volume (C_{70})	**4954.40**		**4926.85**		**4916.94**

Total volume (H_2O---HX)	**251.36**		**293.67**		**310.13**
		Isolated H_2O---HX (X = F, Cl, Br)			
**O1**	125.25	O1	126.14	O1	126.53
**H2**	23.68	H2	23.61	H2	23.83
**H3**	23.64	H3	23.63	H3	23.83
**H5**	10.39	H5	24.89	H4	32.02
**F4**	119.72	Cl4	255.36	Br5	292.41
**Total volume (H_2O---HX)**	**302.68**		**453.63**		**498.62**

Table S7. PBE **c**omputed mean polarizability α (in a.u.) and volume *v* (a.u.) for isolated and complexed C_{70}. Included are also the mean polarizability of isolated dimers.

**Species**	α	***v***	**Isolated**	α	Δα[a]
Free C_{70}	637.51	5055.83		---	
$H_2O\cdots HF@C_{70}$	640.42	5205.76	$H_2O\cdots HF$	10.23	-7.32
$H_2OH^+\cdots{}^-Cl@C_{70}$	643.62	5220.52	$H_2O\cdots HCl$	18.35	-12.24
$H_2OH^+\cdots{}^-Br@C_{70}$	644.82	5227.07	$H_2O\cdots HBr$	23.47	-16.16

[a] Δα(guest@C_{70}) = α(guest@C_{70}) – (α(guest) + α(C_{70}))

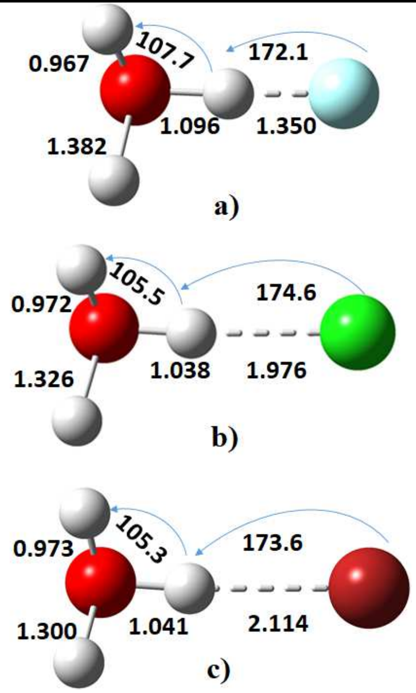

Figure S10. Simulated TD-DFT (PBE/6-311G**) geometries of the $H_2O\cdots HF$, $H_2OH^+\cdots{}^-Cl$ and $H_2OH^+\cdots{}^-Br$ dimers in the first excited electronic state. Selected bond distances and angles are shown in Å and deg, respectively.

TEXT S1 The geometry of isolated $H_2O\cdots HX$ (X = F, Cl, Br) dimers in their first excited and in their anionic ground states.

We compared the geometries of the $H_2O\cdots HX$ (X = F, Cl, Br) dimers in C_{70} and those of the isolated dimers in their respective excited states. For this, the TD-DFT geometries of all three isolated dimers were obtained in their first excited state and are local minima (Fig. S10). The results suggest that the breaking of the HX acids into the H^+ and X^- ions and subsequent formation of the OH_3^+ ion on complete concerted proton transfer are the eventual consequence. The HX acids and the H_2O base are now transformed to the conjugate acid base pairs $[X]^-$ and $[OH_3]^+$, and are linked with each other through a $X^-\cdots{}^+H(OH_2)$ hydrogen bond. The three OH bonds and the HOH bond angles are markedly unequal in these geometries. For instance, two of the OH bonds are virtually identical and the remaining one is markedly longer in all three dimers. The main difference between these geometries and those in C_{70} is that the O–H moieties participating in the hydrogen bond with the halide anion in these have strikingly different bond distances and charge polarities (see Fig. S10 and Fig. 1b-c, for example).

We have also supplied an external electron to the dimers of $H_2O\cdots HX$ (X = F, Cl, Br) dimers, as was done in a previous anion photoelectron spectroscopy and *ab initio* study for $H_3NH^+\cdots{}^-Cl$.[23] Whilst the energy-minimized geometry provided no evidence of proton transfer in the anionic $H_2O\cdots HF$ dimer, the intermolecular distance was indeed significantly reduced. For instance, $r(O\cdots H)$ and $r(O\cdots F)$ in the anionic dimer were 1.519 and 2.525 Å, respectively, and the $\angle O\cdots H–F$ was 176.0°; these signify a similar pattern of intermolecular bonding compared with that observed for the neutral $H_2O\cdots HF$ dimer in C_{70} (Fig. 1a). For $H_2O\cdots HX$ (X = F, Cl, Br), however, the excess electron promoted concerted proton transfer from the acid, and transformed the geometries of the dimers into $H_2OH^+\cdots{}^-Cl$ and $H_3OH^+\cdots{}^-Br$ ion-pair salts. The changes in geometry on the formation of the $H_2O\cdots HF$, $H_2OH^+\cdots{}^-Cl$ and $H_3OH^+\cdots{}^-Br$ anionic dimers are similar to those of the dimers inside the C_{70} cage. However, for the formation of such latter geometries the cage does not supply an external electron to the dimers that effectuates concerted proton transfer between H_2O and HX (X = Cl, Br).

Text T1.

An important feature of any fullerene system lies in the assessment of its binding affinity with the endohedral species. We have used the PBE/6-311G** optimized geometry, and have

calculated on it the basis set superposition corrected binding energy of the $H_2O\cdots HF@C_{70}$ system. In doing so, the entire encaged species $H_2O\cdots HF$ and C_{70} were considered as two monomers of the $H_2O\cdots HF@C_{70}$ supramolecular system, and D3 refers to the D3 version of Grimme's dispersion with the original D3 damping function implemented in Gaussian 09. Although the PBE method underestimates the value, all other functionals provided large values for the binding energy: PBE (+3.78 kcal mol^{-1}), ωB97XD (–16.38 kcal mol^{-1}), B97D3 (–14.65 kcal mol^{-1}), PBE-D3 (–13.41 kcal mol^{-1}), PBE0-D3 (–15.97 kcal mol^{-1}), M06-2X (–21.07 kcal mol^{-1}) and M06-2X-D3 (–22.45 kcal mol^{-1}). These results enable us to demonstrate that binding between C_{70} and the encaged species is reasonably strong; that dispersion is a major driving force responsible for complex stability; and that the widely used M06-2X functional is probably no good as it significantly overestimates the energy of interaction. Nevertheless, our calculation with B97D3 gave uncorrected (and corrected) binding energies of –21.12 (–12.99) and –14.02 (–5.89) kcal mol^{-1}) for $H_2OH^+\cdots{}^-Cl@C_{70}$ and $H_2OH^+\cdots{}^-Br@C_{70}$, respectively.
